# A new genus of Micromygalinae (Araneae, Microstigmatidae) from Brazil, with transfer of *Masteriaemboaba* Pedroso, Baptista & Bertani, 2015 and description of six new species

**DOI:** 10.3897/zookeys.814.29906

**Published:** 2019-01-08

**Authors:** Victor Passanha, Igor Cizauskas, Antonio D. Brescovit

**Affiliations:** 1 Laboratório Especial de Coleções Zoológicas, Instituto Butantan. Av. Vital Brazil, 1500, CEP 05503-900, São Paulo, SP, Brazil Laboratório Especial de Coleções Zoológicas, Instituto Butantan São Paulo Brazil

**Keywords:** mygalomorph, biospeleology, troglobite, Neotropical region, taxonomy

## Abstract

The family Microstigmatidae is composed of two subfamilies, Microstigmatinae and Micromygalinae, seven genera and 16 species. Micromygalinae is monotypic, comprising the species *Micromygalediblemma* Platnick & Forster, 1982 from Panama. A new genus, *Tonton* is described as a new member of the Micromygalinae. *Masteriaemboaba* Pedroso, Baptista & Bertani, 2015, is transferred to the new genus and six new species from Brazil are described and attributed to *Tonton***gen. n**.: the type species, *T.itabirito***sp. n.**, *T.queca***sp. n.**, *T.matodentro***sp. n.** and *T.sapalo***sp. n.**, all from the state of Minas Gerais; *T.ipiau***sp. n.** from the state of Bahia and *T.quiteria***sp. n.** from the state of Maranhão. Among the cavernicolous species, only *T.itabirito***sp. n.** is considered troglobitic by the total absence of eyes.

## Introduction

The spiders of the family Microstigmatidae are known to be small in size, with the exception of *Xenonemesiaplatensis*, which can reach about 10 mm (Goloboff 1989). They can be found living in the leaf litter of Central and South America, and South Africa. Currently the family is divided into seven genera and 17 species (World Spider Catalog 2018), across two subfamilies, Microstigmatinae Roewer, 1924 and Micromygalinae Platnick & Forster, 1982 (Raven and Platnick 1981, Platnick and Forster 1982, Griswold 1985).

Microstigmatinae, after the phylogenetic analysis of Platnick and Forster (1982) resulted in two tribes, both monophyletic: 1. Pseudonemesiini Caporiacco, 1955, including *Pseudonemesia* Caporiacco, 1955 from Colombia and Venezuela and subsequently *Envia* (proposed later the analysis by Ott and Höfer (2003) from Brazil); 2. Microstigmatini with the genera *Microstigmata* Strand, 1932 from South Africa, *Ministigmata* Raven & Platnick, 1981 from Brazil, *Spelocteniza* Gertsch, 1982 from Ecuador, and *Xenonemesia* Goloboff, 1989 from Brazil, Uruguay, and Argentina, the latter two included subsequently and not yet phylogenetically tested.

The subfamily Micromygalinae was proposed by Platnick and Forster (1982) sharing two of the three Microstigmatidae synapomorphies known at the time. The type genus proposed was *Micromygale* with the type species *M.diblemma*, a very small species collected in Panama. Currently, four autoapomorphic characters at least support the monophyly of Micromygalinae: the loss of all but two eyes, the presence in males of an anterior scutum so extensive as to cover most of the abdominal dorsum, the absence of book lungs, and teeth clustered into transverse series of two to nine teeth (Platnick and Forster 1982, fig. 1). Interestingly, this subfamily was elevated to Micromygalidae by Wunderlich (2004), although this nomenclatural modification was not accepted by subsequent authors (Platnick 2014, World Spider Catalog 2018).

As pointed out in Platnick and Forster (1982), small mygalomorph spiders are very abundant in the leaf litter, of which small diplurids of the subfamily Masteriinae stand out. Due to their small size, they may be confused with tiny species of other mygalomorph families, if not examined with great discretion. Recently, in a review of the subfamily Masteriinae Simon, 1889 (Passanha and Brescovit 2018) we examined specimens of *Masteriaemboaba* described by Pedroso et al. (2015) from the type locality. To our surprise these specimens possessed a scaly cuticle (Fig. 2E–F), elevated tarsal organ, oval spiracles and a distinctive serrula (Fig. 12B). All characters found in these spiders can be found in representatives of the family Microstigmatidae.

This study aims to describe a new genus, *Tonton* gen. n., belonging to the subfamily Micromygalinae, increasing its diversity, previously composed of only the genus *Micromygale*. The new genus *Tonton* includes six new species and the previously described, and transferred herein, *Tontonemboaba* (Pedroso et al., 2015). All the species included here are described with comparative diagnoses, illustrations of diagnostic structures, and distribution maps for all species.

## Material and methods

The material examined was deposited in the following Brazilian institutions (curators in parentheses): Instituto Butantan, São Paulo (IBSP, A.D. Brescovit); Universidade Federal de Lavras, Lavras (UFLA, R.L. Ferreira); Universidade Federal de Minas Gerais, Belo Horizonte (UFMG, A.J. Santos). The type material of *Tontonemboaba*, was deposited by the authors in the MNRJ; due to a fire in the collection it was not possible to examine them, probably all the types were lost, but material of the type locality was examined. All measurements are in millimeters and were obtained by using an ocular lens with a micrometer scale. Total body size was measured without spinnerets and chelicerae. Width and length of carapace, eye tubercle, labium and sternum are the maximum values. Size of leg segments was measured between the joints in lateral view.

Illustrations were made using a stereomicroscope with camera lucida. For illustrating female genitalia, we used dissected organs; soft tissues were digested for 72 hours using Ultrazyme® enzymatic contact lens cleaner diluted in distilled water. Image stacks were obtained with a Leica DM2500. Female spermathecae were photographed in temporary lamina.

Material for SEM was either air-dried or dehydrated using an ethanol series followed by immersion in HDMS. All material for SEM was fixed to stubs with double-faced adhesive copper tape and sputter-coated with gold. SEM images were taken using a Quanta 250 Scanning Electron Microscopy with an attached SLR digital camera at Institute Butantan. The descriptions follow Platnick and Forster (1982), with nomenclatural modifications in some terminologies. The spination of the legs follows the model proposed by Petrunkevitch (1925).

Geographic coordinates were obtained using Google Earth (Lat/Long – WGS84) and species distribution maps were made using the DIVA-GIS 7.5 program.

## Taxonomy

### Family Microstigmatidae Roewer, 1942

#### Subamily Micromygalinae Platnick & Forster, 1982

##### Tonton gen. n.

Taxon classificationAnimaliaAraneaeMicrostigmatidae

http://zoobank.org/A5EF11F4-E89E-4B3F-8BF5-CCCB85B03F33

###### Type species.

*Tontonitabirito* sp. n.

###### Etymology.

The generic name is derived from the Indian Krenak word “tón-tón”, and means small. The Krenak Indians, also known as Botocudos or Aimorés and inhabited mainly southeastern Brazil, with great occupation in areas of the state of Minas Gerais. The generic name is neuter.

###### Diagnosis.

**Figure 1.**
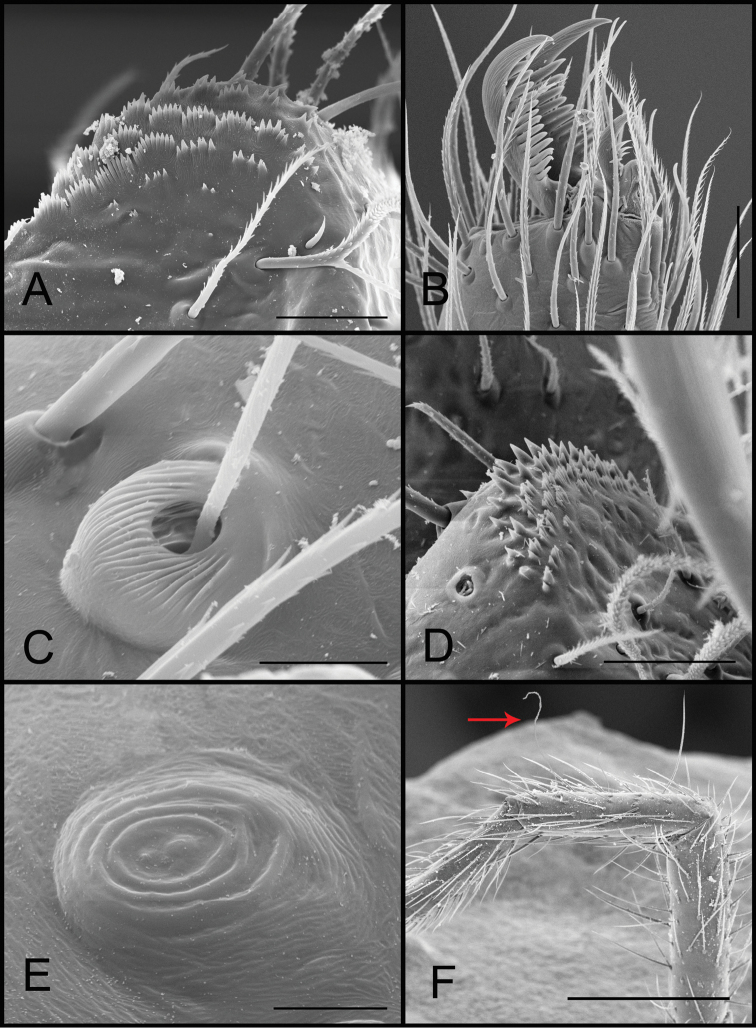
*Tonton* spp. morphological characters. **A** serrula, lateral view **B** tarsal claw, retrolateral view **C** tricobothrium, dorsal view **D** detail of serrula and projected palpal coxae, ventral view **E** tarsal organ, dorsal view **F** detail of slender tricobothria, leg I. Scale bars: 20 μm (**A**); 30 μm (**B**); 5 μm (**C**); 20 μm (**D**); 5 μm (**E**); 300 μm (**F**).

**Figure 2.**
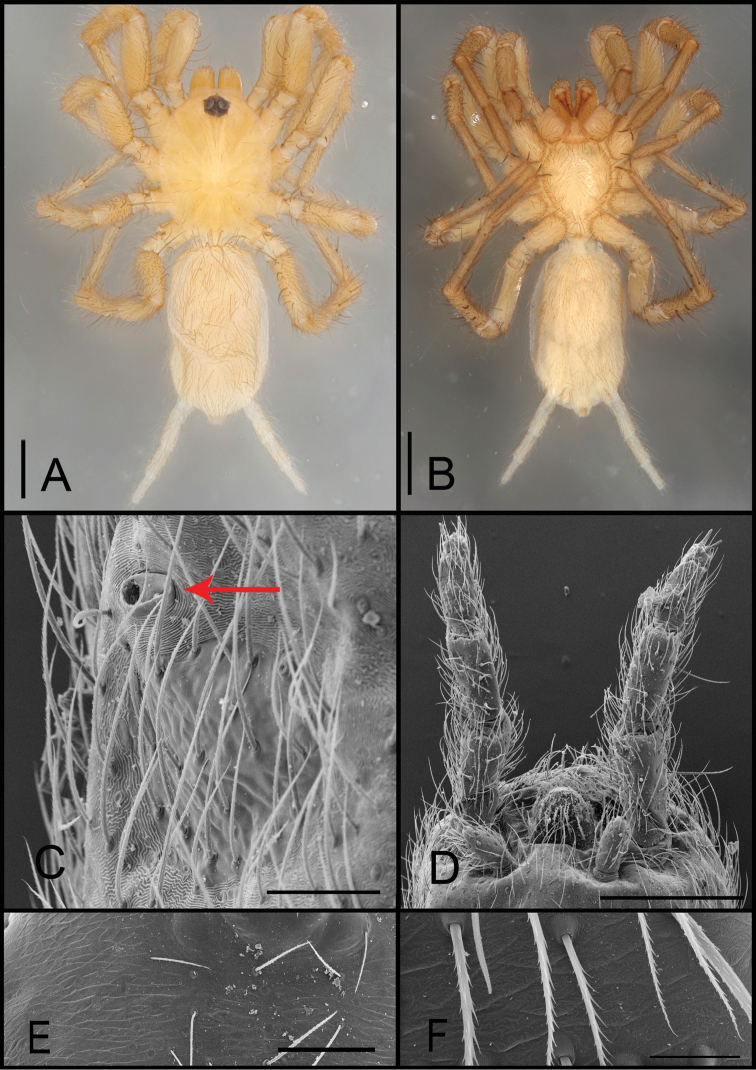
*Tonton* spp. morphological characters. **A** body coloration in dorsal view (*T.ipiau* sp. n.) **B** body coloration in ventral view (*T.ipiau* sp. n.) **C** booklung opening, ventral view (*T.ipiau* sp. n.) **D** spinnerets, ventral view (*T.ipiau* sp. n.) **E, F** detail of cuticle in *T.emboaba* (cephalothorax, dorsal view and Leg I, prolateral view). Scale bars: 1**m**m (**A**); 1**m**m (**B**); 50µm (**C**); 200µm (**D**); 100µm (**E**); 200µm (**F**).

Species of the genus *Tonton* differ from those of *Micromygale*, the only representative of Micromygalinae (see Platnick and Forster 1982: figs 12–15, 23–26), by having four spinnerets (Fig. 2B, D), while *Micromygale* have six (see Platnick and Forster 1982: fig. 24), lack a paraembolic apophysis on male palpal bulb (Fig. 1F), present in *Micromygale* (see Platnick and Forster 1982: figs 12–15), having bilobed spermathecae (Fig. 4E–F), in *Micromygale* unilobed (see Platnick and Forster 1982: figs 27–28), having a pulmonar opening (Fig. 2C), absent in *Micromygale*, and the lack of an abdominal shield (Fig. 2B), present in *Micromygale* (see Platnick and Forster 1982: fig. 23).

###### Description.

**Figure 3.**
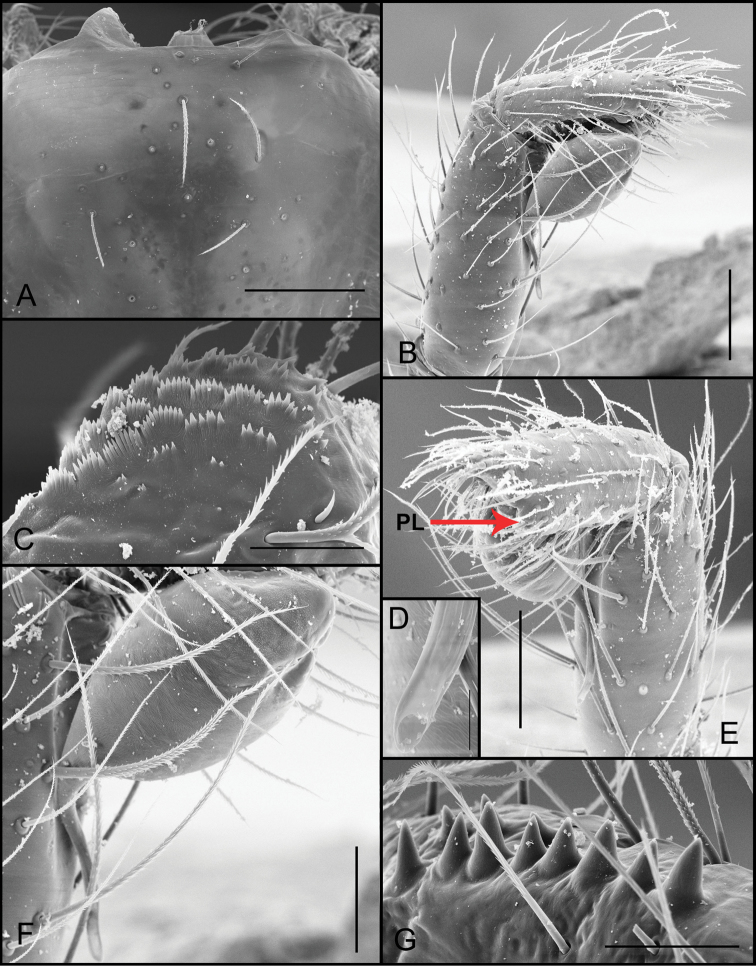
*Tontonitabirito* sp. n. (male, Itabirito, Minas Gerais, IBSP 196177), **A** detail of cephalic area of carapace **B** palp, retrolateral view **C** serrula, lateral view **D** embolous tip, ventral view **E** palp, prolateral view; arrow indicates prolateral lobe (PL) **F** bulb, retrolateral view **G** chelicerae teeth, prolateral view. Scale bars: 100 μm (**A**); 100 μm (**B**); 20 μm (**C**); 10 μm (**D**); 100 μm (**E**); 50 μm (**F**); 30 μm (**G**).

**Figure 4.**
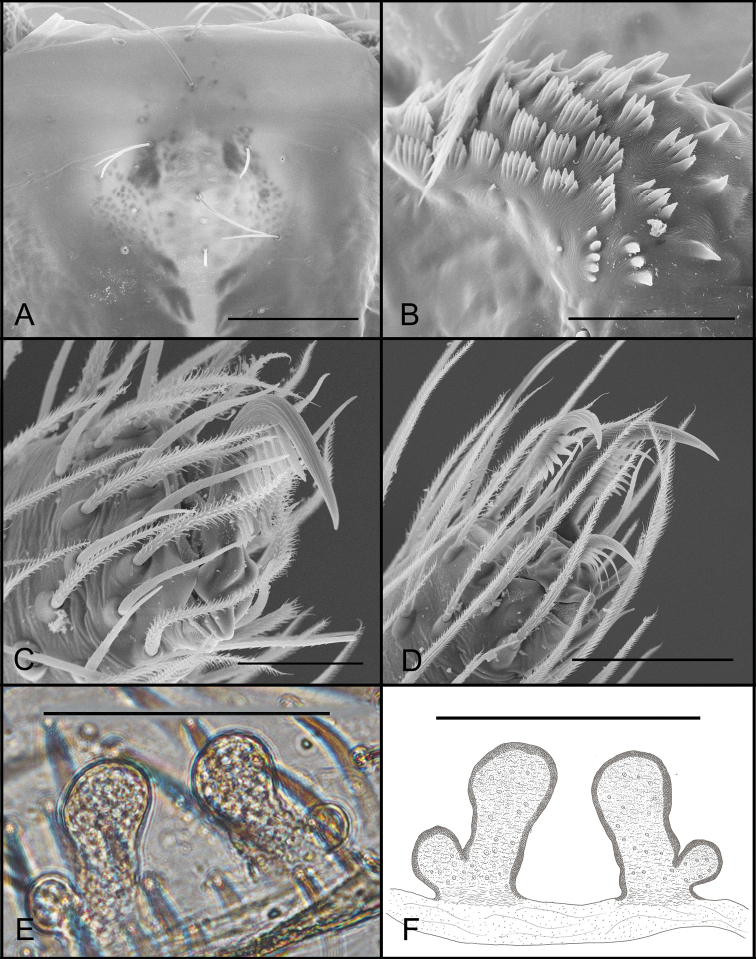
*Tontonitabirito* sp. n., (female, Itabirito, Minas Gerais, IBSP 196177), **A** detail of cephalic area of carapace **B** serrula, lateral view **C** palpal claw, lateral view **D** tarsal claws, left leg I **E, F** female genitalia, dorsal view. Scale bars: 100 μm (**A**); 20 μm (**B**); 30 μm (**C**); 50 μm (**D**); 100 μm (**E, F**).

**Figure 5.**
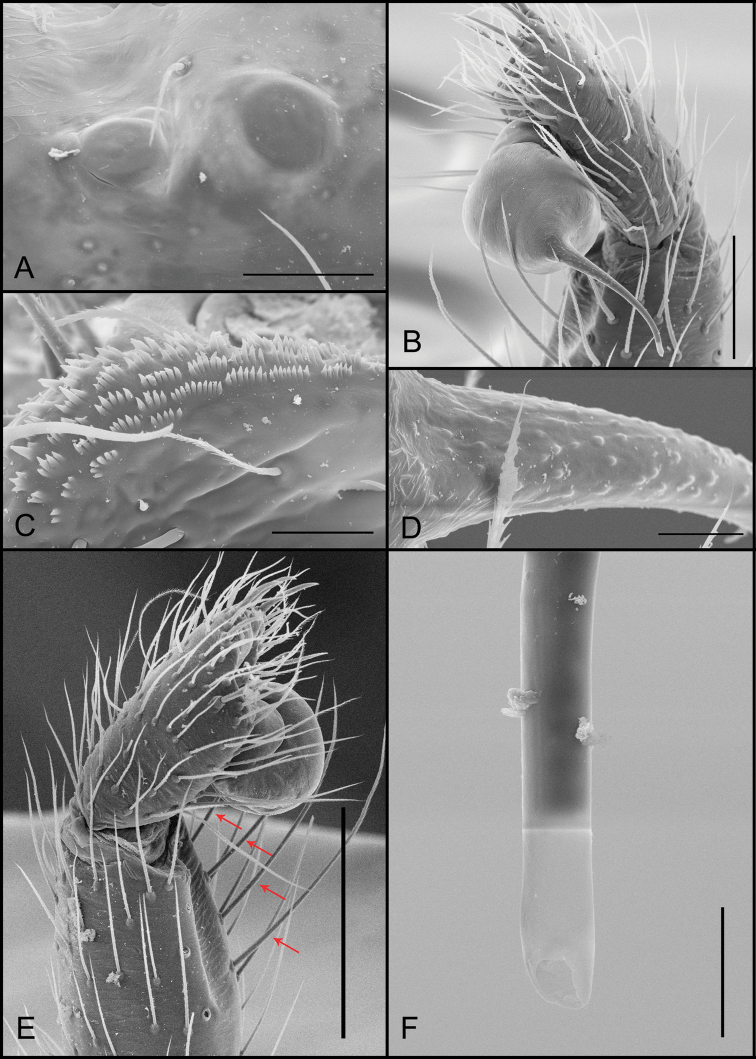
*Tontonqueca* sp. n. (male, Itabirito, Minas Gerais, IBSP 97956), **A** eyes, dorsal view **B** palp, retrolateral view **C** serrula, lateral view **D** embolous base, ventral view **E** palp, prolateral view (arrows indicate row of setae) **F** embolous tip, ventral view. Scale bars: 100 μm (**A**); 100 μm (**B**); 20 μm (**C**); 10 μm (**D**); 200 μm (**E**); 10 μm (**F**).

**Figure 6.**
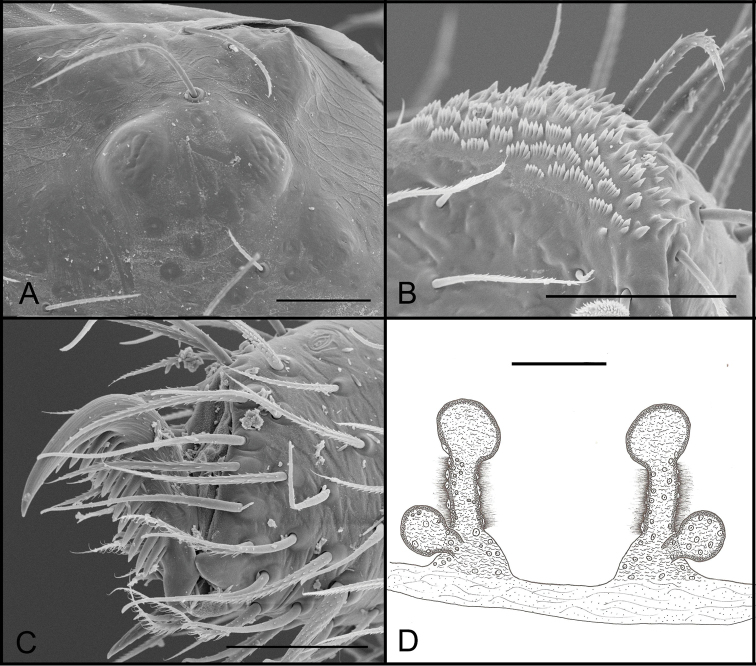
*Tontonqueca* sp. n. (female, Itabirito, Minas Gerais, IBSP 97963), **A** eyes, dorsal view **B** serrula, lateral view **C** palpal claw, prolateral view **D** female genitalia, dorsal view. Scale bars: 100 μm (**A**); 50 μm (**B, C**); 10 μm (**D**).

**Figure 7.**
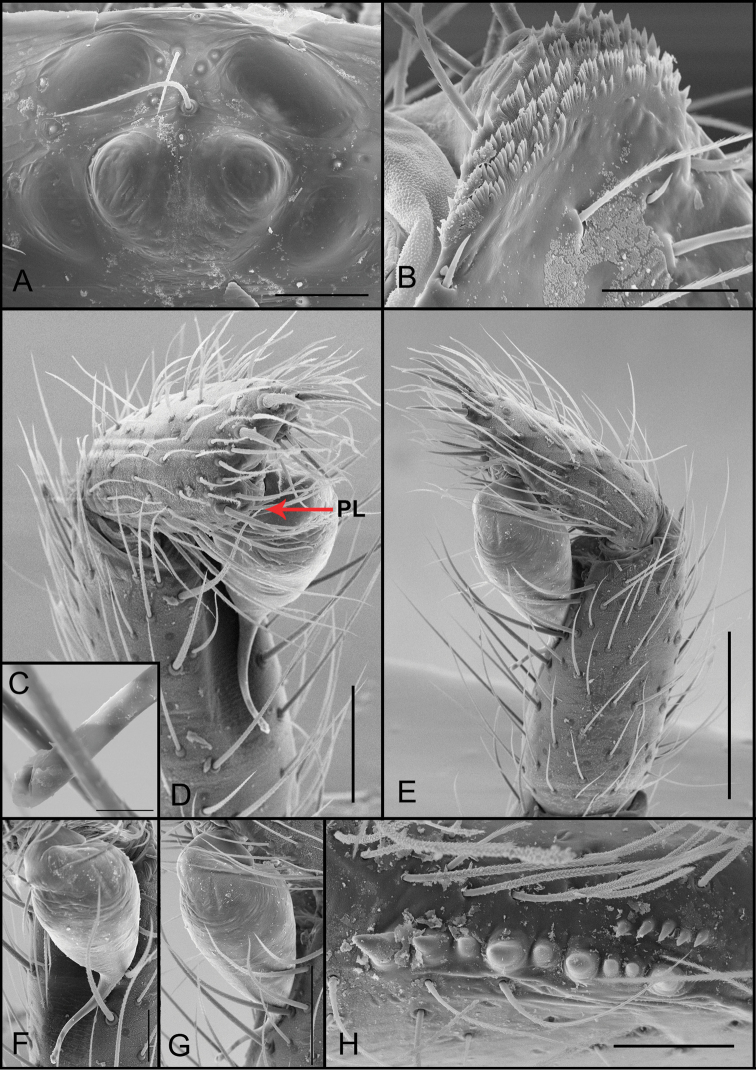
*Tontonmatodentro* sp. n. (male, Conceição do Mato Dentro, Minas Gerais, IBSP 196239), **A** eyes, dorsal view **B** serrula, lateral view **C** embolous tip, ventral view **D** palp, prolateral view; arrow indicate prolateral lobe (PL) **E** palp, retrolateral view **F, G** bulb, retrolateral view, **H** chelicerae teeth, ventral view. Abbreviations: PL, prolateral lobe. Scale bars: 50 μm (**A**); 30 μm (**B**); 100 μm (**C**); 200 μm (**D, E**); 50 μm (**F, G, H**).

Small-sized mygalomorph spiders, total length 1.80–3.04. Coloration: carapace in dorsal view, chelicerae, legs varying from whitish to light yellow. Palpal coxae, labium, sternum whitish to light yellow. Abdomen whitish or light yellow (Fig. 2A, B). Clypeus narrow (0.02–0.06), absent in *T.itabirito* sp. n. Chelicerae without rastellum. Basal segment of chelicerae with prolateral row of 6–14 teeth, 4–10 mesobasal teeth aligned (Fig. 7H). Absence of ocular tubercle, with six eyes (lateral eyes vestigial) (Fig. 6A) or absence of eyes, having only a vestigial ocular spot (*T.itabirito* sp. n.) (Fig. 4A); anterior/posterior row slightly recurved, AME absent. Labium wider than long, without cuspules. Palpal coxae with short anterior lobe, bearing serrula with teeth clumped in series of several rows on distal border (Fig. 1A, D). Sternum oval, longer than wide, without visible sigilla. Legs with one row of tricobothria on tarsi/metatarsi (dorsal); two on dorsal tibia. Tarsus without scopulae, with sensorial setae thin, slightly interspaced or thick, abundant. Superior tarsal claws with one row of teeth (6–14), inferior tarsal claw present on all legs, with 1–5 teeth (Fig. 1B). Tricobothria filiform, with corrugated base, with keels until half of extension (Fig. 1C, F). Abdomen with ovate pulmonar opening (Fig. 2C). Posterior median spinneret short with spigots on apex. Posterior lateral spinneret three-segmented, long, apical segment with triangular apex, spigots present on ventral face (major ampulate, aciniform, and pumpkiniform spigots) (Fig. 2D). Female palp varying from 2–7 ventral spines tarsal basis, tarsal claw with one central row of 11–15 teeth (Fig. 6C). Male palp: cymbium with 4–6 apical spines (Fig. 7D), prolateral lobe (Figs 3E, 7D) and one row of trichobothria on tarsi and two on tibia; palpal tibia with one discreet row of retrolateral setae, on medial region (Fig. 5E), excavated in distal median area (Fig. 5E); piriform bulb (oval in *T.queca* sp. n.); elongated embolus almost length of the bulb (Fig. 10C). Female genitalia: two spermathecae, with two lobes each side, internal lobe long, external lobe short (Fig. 6D), except *T.ipiau* sp. n., almost as long as external lobe (Fig. 10F–G).

###### Distribution.

Cave and mountainous region of the state of Minas Gerais and states of Bahia (Atlantic forest) and Maranhão (Brazilian cerrado).

##### Tonton itabirito sp. n.

Taxon classificationAnimaliaAraneaeMicrostigmatidae

http://zoobank.org/3C8D862F-EA9D-4A42-B460-6E24143C7567

Figures 3 , 4 , 13

###### Types.

Holotype male and paratype female from Itabirito (20°14'30.0"S, 43°48'51.1"W), Minas Gerais, no data, Marcus leg., deposited in IBSP196178 and IBSP196179.

###### Other material examined.

BRAZIL. **Minas Gerais**: Barão de Cocais, Cave RF-083 (19°57'23"S, 43°35'15"W), Am. 28, 6♀, 10–21.III.2009, R. Andrade et al. leg. (IBSP 196176); Itabirito, Cave CPTM_14 (20°7'1"S, 43°53'56"W), Am. 22, 1♂ 2♀ 2 imm., 09.I.2013, M.P.A. de Oliveira leg. (IBSP 196177); Cave CPTM_15 (20°7'1"S, 43°53'55"W), Am. 12, 1♀ 5 imm., 09.I.2013, M.P.A. de Oliveira leg. (IBSP 196187); Cave CPTM_08 (20°7'25"S, 43°54'5"W), Am. 36, 2♀, 14.I.2013, M.P.A. de Oliveira leg. (IBSP 196188); Rio Acima, Cave VG-29 (20°7'5"S, 43°53'40"W), Am. 262, 1♀, 29.III–01.IV.2011, R. Andrade & I. Cizauskas et al. leg. (IBSP 196180); Cave VG-27 (20°7'0"S, 43°53'53"W), Am. 88, 1♀, 29.III–01.IV.2011, R. Andrade & I. Cizauskas et al. leg. (IBSP 196181); Cave VG-20, (20°7'23"S, 43°54'3"W), Am. 53, 2♀, 29.III–01.IV.2011, R. Andrade & I. Cizauskas et al. leg. (IBSP 196182); Cave VG-27 (20°7'0"S, 43°53'53"W), Am. 250, 1♀, 2–10.VIII.2011, R. Andrade & I. Cizauskas et al. leg. (IBSP 196183); Cave VG-27 (20°7'0"S, 43°53'53"W), Am. 249, 1♀, 2–10.VIII.2011, R. Andrade & I. Cizauskas et al. leg. (IBSP 196184); Cave VG-27 (20°7'0"S, 43°53'53"W), Am. 247, 1♀, 2–10.VIII.2011, R. Andrade & I. Cizauskas et al. (IBSP 196185); Cave VG-26 (20°7'0"S, 43°53'54"W), Am. 232, 2♀, 2–10.VIII.2011, R. Andrade & I. Cizauskas et al. leg. (IBSP 196186); Cave VG-01 (20°9'9"S, 43°49'0"W), 1♂, 2–10.VIII.2011, I. Cizauskas leg. (IBSP 198496; MEV); Cave VG-01 (20°9'9"S, 43°49'0"W), Am. 01, 1♀, 29.III–01.V.2011 (IBSP 196197); Cave VG-01 (20°9'9"S, 43°49'0"W), Am. 02, 4♀, 29.III–01.V.2011 (IBSP 196199); Cave VG-01 (20°9'9"S, 43°49'0"W), Am. 153, 1♀, 29.III–01.V.2011 (IBSP 196200), all collected by R. Andrade & I. Cizauskas et al.

###### Etymology.

The specific epithet is a noun taken in apposition and refers to the type locality.

###### Diagnosis.

Males of *Tontonitabirito* sp. n. resemble the males of the other species in the genus by the piriform bulb, but differ by the absence of eyes (Fig. 3A). Females are similar to *T.quiteria* sp. n. by the spermathecae with the thickened internal lobe (Fig. 11E), but differ by the uniform apex (Fig. 4E–F).

###### Description.

**Male (holotype).** Coloration: carapace and legs whitish, abdomen yellowish. Eyes absence, strong ocular spot (Fig. 3A). Total length 1.98. Carapace 0.9 long, 0.68 wide. Abdomen 1.08 long. Fovea 0.12. Labium 0.13 long, 0.18 wide. Sternum 0.50 wide, 0.65 long. Serrula with three rows of teeth clumped in series (Fig. 3C). Palp: femur 0.53/ patella 0.23/ tibia 0.38/ tarsus 0.25/ total 1.39. Legs: I femur 0.76/ patella 0.34/ tibia 0.64/ metatarsus 0.42/ tarsus 0.34/ total 2.50; II 0.63/ 0.32/ 0.5/ 0.35/ 0.32/ 2.12; III 0.65/ 0.29/ 0.45/ 0.5/ 0.39/ 2.28; IV 0.82/ 0.33/ 0.62/ 0.64/ 0.46/ 2.87. Leg formula 4132. Spination: palp: tarsus d4ap; leg I patella v1, tibia v2-2ap, metatarsus v1-2ap; II patella v2, tibia v2-3ap, r1, metatarsus v2-3ap, p1; III patella v2, p1, r1, tibia d2-2ap, p2, r3, metatarsus d2, v3-3ap, p2, r2; IV patella p1, r1, tibia d2, v1-3ap, p2, r3, metatarsus d2, v3ap, p1, r1. Tibia I with two or three apical ventral spines. Palpal tibia two or three times the size of cymbium, with distal area of bulb resting in a ventral depression. Cymbium as long as wide with a short prolateral lobe (Fig. 3E), with four apical spines (Fig. 3B, E). Bulb piriform, embolus with the same size of tegulum, with discrete median sinuosity, apex of embolus slightly rounded (Fig. 3B, D–F). PLS: basal, medial and apical segments, 0.24, 0.18, 0.23 long.

**Female (Paratype).** Coloration as in male. Eyes and strong ocular spot as in male (Fig. 4A). Total length 2.03. Carapace 0.82 long, 0.58 wide. Abdomen 1.20 long. Fovea 0.12. Labium 0.05 long, 0.13 wide. Sternum 0.40 wide, 0.55 long. Serrula with four rows of teeth clumped in series (Fig. 4B). Palp: femur 0.46/ patella 0.24/ tibia 0.29/ tarsus 0.33/ total 1.32; leg I femur 0.65/ patella 0.3/ tibia 0.43/ metatarsus 0.34/ tarsus 0.27/ total 1.99; II 0.48/ 0.25/ 0.3/ 0.26/ 0.25/ 1.54; III 0.46/ 0.24/ 0.3/ 0.25/ 0.29/ 1.54; IV 0.58/ 0.26/ 0.43/ 0.41/ 0.37/ 2.05. Leg formula 4123. Spination: leg III tibia r1, metatarsus d2, p1, r1; IV tibia v2, p2, r2, metatarsus v2-2ap, p1, r1. Genitalia with two bilobed spermathecae, internal lobe thickened with rounded and uniform apex, external lobe short and wide, glands in both lobes, scattered irregularly and enlarged genitalia basis, (Fig. 4E, F). PLS: basal, medial and apical segments, 0.47, 0.63, 0.47 long.

###### Natural history.

*Tontonitabirito* sp. n. is the only anophthalmic troglobite species here described. The specimens were collected mainly in iron caves in the Quadrilátero Ferrífero Iron Region, in deep regions on the cave floor.

###### Distribution.

In the cities of the Rio Acima, Itabirito, Barão do Cocais, in the state of Minas Gerais (Fig. 13).

##### Tonton queca sp. n.

Taxon classificationAnimaliaAraneaeMicrostigmatidae

http://zoobank.org/B65A7DE9-5279-4C22-A811-F7866ACB14EA

Figures 5 , 6 , 13

###### Types.

Holotype male and paratype female from Nova Lima, Cave MS-150 (20°12'6"S, 43°58'1"W), Am. 66, 1♂ 1♀, 28.X.2005, R.L. Ferreira leg., deposited in IBSP 198537.

###### Other material examined.

BRAZIL. **Minas Gerais**: Itabirito, Cave VL-23 (20°20'10"S, 43°56'1"W), Am. 149, 1♂ 3 imm., 3–20.XI.2007, R. Andrade leg. (IBSP 97956; pictures); Cave VL-12 (20°17'47"S, 43°56'48"W), Am. 119, 3♀, 3–20.XI.2007, R. Andrade leg. (IBSP 97963, pictures); Cave VL-25 (20°20'9"S, 43°56'5"W), Am. 142, 5♀ 1 imm., 3–20.XI.2007, R. Andrade leg. (IBSP 97955); Cave VL-35 (20°18'29"S, 43°56'33"W), Am. 161, 1♀, 3–20.XI.2007, R. Andrade leg. (IBSP 97945); Mina do Pico, Cave (20°13'15"S, 43°52'56"W), Am. 34, 1♀, 31.V.2013, BioEspeleo Cons. Amb. leg. (IBSP 196191), external collect, in the forest; Mina do Pico Cave (20°13'15"S, 43°52'56"W), Am. 33, 5♀ 5 imm., 10.VI.2013, BioEspeleo Cons. Amb. leg. (IBSP 196191), external collect, in the forest; Mina do Pico Cave (20°13'15"S, 43°52'56"W), Am. 28, 2♀, 05.VI.2013, BioEspeleo Cons. Amb. leg. (IBSP 196191), external collect, in the forest; Mina do Pico Cave (20°13'15"S, 43°52'56"W), Am. 30, 1♀ 1 imm., 5.VI.2013, BioEspeleo Cons. Amb. leg. (IBSP 196191), external collect, in the forest; Itabirito, Cave MP-004 (20°13'22"S, 43°51'13"W), Am. 64, 1♀, 31.VIII.2005, (IBSP 198533); Cave MP-008 (20°12'39"S, 43°51'12"W), Am. 63, 5♀, 08.IX.2005 (IBSP 198538); Cave MP-009 (20°12'39"S, 43°51'15"W), Am. 65, 1♀, 01.IX.2005 (IBSP 198539); Cave MP-014 (20°13'41"S, 43°51'47"W), Am. 67, 1♀, 30.VIII.2005 (IBSP 198540); Cave MP-005 (20°13'21"S, 43°51'14"W), Am. 60, 1♀, 01.IX.2005 (IBSP 198541); Cave MP-007 (20°12'41"S, 43°51'12"W), Am. 69, 1♀, 01.IX.2005 (IBSP 198542); Cave MP-011 (20°13'4"S, 43°51'26"W), Am. 68, 2♀, 07.IX.2005 (IBSP 198543), all collected by R.L. Ferreira; Rio Acima, Cave VG-02 (20°9'29"S, 43°49'8"W), Am. 04, 1♀, 29.III–01.V.2011, R. Andrade & I. Cizauskas et al. leg. (IBSP 196195); Cave VG-02 (20°9'29"S, 43°49'8"W), Am. 157, 1 ♀, 29.III–01.V.2011, R. Andrade & I. Cizauskas et al. leg. (IBSP 196196); Cave VG-20 (20°7'23"S, 43°54'3"W), Am. 212, 1♀, 29.III–01.V.2011 (IBSP 196198); Cave VG-20 (20°7'23"S, 43°54'3"W), Am. 58, 2♀, 29.III–01.V.2011 (IBSP 196201); Cave VG-05 (20°6'5"S, 43°53'10"W), Am. 164, 2♀, 29.III–01.V.2011 (IBSP 196202); Cave VG-05 (20°6'5"S, 43°53'10"W), Am. 14, 1♀, 29.III–01.V.2011 (IBSP 196203); Cave VG-29 (20°7'5"S, 43°53'40"W). Am. 262, 1♀, 29.III–01.V.2011 (IBSP 196204); Cave VG-06 (20°9'30"S, 43°49'8"W), Am. 06, 4♀, 29.III–01.V.2011 (IBSP 196205), all collected by R. Andrade & I. Cizauskas et al. leg.; Nova Lima, Cave MS-150 (20°12'6"S, 43°58'1"W), Am. 62, 1♀ 1 imm., 04.II.2005, (IBSP 198534); Cave MS-150 (20°12'6"S, 43°58'1"W), Am. 66, 1♂ 5♀ 3 imm., 28.X.2005 (IBSP 198535); Cave MS-150 (20°12'6"S, 43°58'1"W), Am. 66, 1♂, 28.X.2005 (IBSP 198536; SEM), all collected by R.L. Ferreira; Igarapé, Morro do Ipe, Cave_01 (20°6'26"S, 46°11'44"W), Am. 27, 1♀, 23.I.2018, BioEspeleo Cons. Amb. leg. (IBSP 198544).

###### Etymology.

The specific name refers to queca, a cake of English origin, brought to Nova Lima at the time of gold exploration. It became part of the traditional cuisine, as a dessert in the Christmas period and today considered a material good of the municipality.

###### Diagnosis.

Specimens of the *T.queca* sp. n. can be distinguished from other species by the oval bulb and slender embolus with micro-basal projections (Fig. 5B, D). Females resemble *T.sapalo* sp. n. by having a spermathecae with narrow duct of the internal lobe and rounded apex (Fig. 9H), but differ by the larger base and distinct setae in all internal lobe ducts (Fig. 6D).

###### Description.

**Male (holotype).** Coloration: carapace in dorsal and ventral view, and legs whitish, abdomen yellowish. Total length 1.95. Carapace 0.95 long, 0.65 wide. Abdomen 1.0 long. Fovea 0.13. Clypeus 0.03. Eyes: six, the lateral eyes are vestigial, PME 0.02, lateral eyes are not visible to be measured (Fig. 5A). Labium 0.1 long, 0.15 wide. Sternum 0.45 wide, 0.58 long. Serrula with 5–6 rows of teeth clumped in series (Fig. 5B). Palp: femur 0.53/ patella 0.23/ tibia 0.38/ tarsus 0.25/ total 1.39. Legs I femur 0.76/ patella 0.34/ tibia 0.64/ metatarsus 0.42/ tarsus 0.34/ total 2.5; II 0.63/ 0.32/ 0.5/ 0.35/ 0.32/ 2.12; III 0.65/ 0.29/ 0.45/ 0.5/ 0.39/ 2.28; IV 0.82/ 0.33/ 0.62/ 0.64/ 0.46/ 2.87. Leg formula 4132. Spination: palp: tibia r3, tarsus d4ap; leg I: patella v1, tibia v3-2ap, p1, metatarsus v2-2ap, p1; II: patella v1, tibia v3-2ap, p1, metatarsus v3-2ap, p1; III: patella v2, p2, r1, tibia d1, v3-2ap, p2, r1, metatarsus d2, v1-2ap, p2, r2; IV: patella v2, r1, tibia v3-3ap, p2, r2, metatarsus d2, v1-2ap, p2, r2. Tibia I with two apical ventral spines. Palpal tibia two times the size of cymbium, with the bulb resting in a ventral distal depression. Cymbium as long as wide with a short prolateral lobe, with five apical spines (Fig. 5B, E). Embolus filiform, with the same size of tegulum, with discreet medial curvature, with medial micro protuberances, apex of embolus slightly rounded (Fig. 5B, D–F). PLS: basal, medial and apical segments, 0.21, 0.19, 0.18 long.

**Female (paratype).** Coloration as in male. Total length 2.14. Carapace 0.84 long, 0.66 wide. Abdomen 1.3 long. Fovea 0.15. Clypeus 0.02. Eyes as in male, ALE 0.07, PME 0.03, PLE 0.06 (Fig. 6A). Labium 0.1 long, 0.2 wide. Sternum 0.5 wide, 0.68 long. Serrula with rows as in male. Palp: femur 0.65/ patella 0.34/ tibia 0.35/ tarsus 0.34/ total 1.68. Leg I femur 0.78/ patella 0.42/ tibia 0.51/ metatarsus 0.38/ tarsus 0.31/ total 2.4; II 0.64/ 0.34/ 0.38/ 0.33/ 0.3/ 1.99; III 0.63/ 0.46/ 0.46/ 0.34/ 0.31/ 2.2; IV 0.78/ 0.47/ 0.55/ 0.51/ 0.42/ 2.73. Leg formula 4132. Spination: palp, tibia v2-3ap, tarsus v2-2-2; leg I tibia v2-2ap, metatarsus v3-2ap; II patella v2, p2, tibia v2-3ap, p3, metatarsus v3-2ap, p1; III patella v2, p1, tibia v2-2ap, p1, metatarsus v3-2ap, p1; IV patella r1, tibia v1-2ap, p1, r2, metatarsus d2, v1-3ap, p2, r3. Genitalia with external lobe connected to the raised basis and glands of both lobes (Fig. 6D). PLS: basal, medial and apical segments, 0.32, 0.23, 0.25 long.

###### Natural history.

All specimens were collected in the iron caves in the Quadrilátero Ferrífero Iron Region. The specimens were collected on the floor below blocks in aphotic zones of the caves, and they are troglobite species.

###### Distribution.

Nova Lima, Itabirito, Rio Acima and Igarapé, in the state of Minas Gerais (Fig. 13).

##### Tonton matodentro sp. n.

Taxon classificationAnimaliaAraneaeMicrostigmatidae

http://zoobank.org/9BC2A86D-72E4-487C-A47C-2EFF753FFF72

Figures 7 , 8 , 13

###### Types.

Male holotype from Cave SERP 124, Conceição do Mato Dentro, Minas Gerais, Brazil, A. Koken leg., deposited in IBSP 196238. Female paratype from Cave RF 52, 655021mE/ 7795051mN, Barão de Cocais, Minas Gerais, Brazil, deposited in IBSP 196277.

###### Other material examined.

BRASIL. Minas Gerais: Morro do Pilar, Cave SERP_0056 (19°5'33"S, 43°20'42"W), Am. 89, 1♀ 1 imm., 23.VII.2013 (IBSP 196234); Cave SERP_0004 (19°6'14"S, 43°19'58"W), Am. 71, 1♀ 1imm., 26.VII.2013 (IBSP 196235); Cave SERP_0032 (19°2'16"S, 43°23'12"W), Am. 129, 1♀, 06.VII.2013 (IBSP 196236); Cave SERP_0010 (19°2'54"S, 43°23'9"W), Am. 50, 2♀ 1 imm., 30.VII.2013 (IBSP 196237), all collected by A. Koken; Cave Gruta MP-07 (19°15'28"S, 43°22'33"W), Am. 356, 1♀, 2013, A. Koken leg. (IBSP 196245); Conceição do Mato Dentro, Cave SERP_0100 (19°3'1"S, 43°24'11"W), Am. 607, 1♂, 1♀, 03.XII.2013 (IBSP 196239); Cave SERP_0195 (19°5'57"S, 43°20'24"W), Am. 604, 1♀, 03.XII.2013 (IBSP 196240); Cave SERP_0183 (19°5'33"S, 43°20'45"W), Am. 605, 1♀, 22.I.2014 (IBSP 196241); Cave SERP 0033, Am. 614, 1♀, 14.XII.2013 (IBSP 196242); Cave SERP_0135 (19°2'42"S, 43°23'21"W), Am. 616, 1♀, 1 imm., 04.XII.2013 (IBSP 196243), all collected by A. Koken; Cave MP 14 (19°8'14"S, 43°24'36"W), Am. 434, 1♀, 13–17.II.2012 (IBSP 196244); Cave ASS_08, Abrigo Taboão (18°54'17"S, 43°27'15"W), Am. 05, 1♂, 15.XII.2010–14.I.2011 (IBSP 196246); Cave ASS 01 (ex Cave 13) (18°54'21"S, 43°25'48"W), Am. 40, 1♀ 1 imm., 15.XII.2010–14.I.2011 (IBSP 196247), all collected by R. Bessi et. al.; Cave CMN-06 (19°0'6"S, 43°24'30"W), Am. 289, 1 imm., 12.III–02.IV.2014 (IBSP 196248); Cave CSF 05 (ex Cave PT 140) (18°58'34"S, 43°23'55"W), Am. 213, 1♀, 12.III–02.IV.2014 (IBSP 196249); Cave CSF 21 (18°59'26"S, 43°23'34"W), Am. 551, 2 imm., 12.III–02.IV.2014 (IBSP 196250); Cave CSF 05 (ex Cave PT 140) (18°58'34"S, 43°23'55"W), Am. 214, 3♀ 1 imm., 12.III–02.IV.2014 (IBSP 196251); Cave CSF 21 (18°59'26"S, 43°23'34"W), Am. 553, 1♀ 1 imm., 12.III–02.IV.2014 (IBSP 196252); Cave CSS-68 (18°56'34"S, 43°24'43"W), Am. 186, 1♀, 12.III–02.IV.2014 (IBSP 196253); Cave CMN-22 (18°59'39"S, 43°24'27"W), Am. 312, 1♀, 12.III–02.IV.2014 (IBSP 196254); Cave CSF 27 (18°59'20"S, 43°24'1"W), Am. 354, 1♀, 12.III–02.IV.2014 (IBSP 196255); Cave CSF 24 (18°59'30"S, 43°23'51"W), Am. 563, 2♀, 12.III–02.IV.2014 (IBSP 196256); Cave CSF 14 (18°57'15"S, 43°24'7"W), Am. 510, 1♀, 1 imm., 12.III–02.IV.2014 (IBSP 196257); Cave CSF 05 (ex Cave PT 140) (18°58'34"S, 43°23'55"W), Am. 479, 2♀, 23.VI–12.VII.2014 (IBSP 196258; detached epigynum); Cave CSF 05 (ex Cave PT 140) (18°58'34"S, 43°23'55"W), Am. 477, 3♀, 1 imm., 23.VI–12.VII.2014 (IBSP 196259); Cave CSF 24 (18°59'30"S, 43°23'51"W), Am. 342, 1♀, 23.VI–12.VII.2014 (IBSP 196260); Cave CSF 36 (18°59'35"S, 43°23'44"W), Am. 591, 1♀ 1 imm., 23.VI–12.VII.2014 (IBSP 196261); Cave CSF 08 (ex Caverna PT 34) (18°59'30"S, 43°23'52"W), Am. 341, 1♀, 12.III–02.IV.2014 (IBSP 196262); Cave CSF 14 (18°57'15"S, 43°24'7"W), Am. 516, 2♀, 12.III–02.IV.2014 (IBSP 196263); Cave CSF 16 (18°57'32"S, 43°24'6"W), Am. 530, 1♀, 12.III–02.IV.2014 (IBSP 196264); Cave CMN-07 (19°0'9"S, 43°24'33"W), Am. 438, 1♀, 23.VI–12.VII.2014 (IBSP 196265); Cave CMN-22 (18°59'39"S, 43°24'27"W), Am. 313, 1♀, 12.III–02.IV.2014 (IBSP 196266); Cave CSF 05 (ex Cave PT 140) (18°58'34"S, 43°23'55"W), Am. 478, 1♀ 1 imm., 23.VI–12.VII.2014 (IBSP 196267); Cave CSF_50 (18°59'17"S, 43°23'54"W), Am. 983, 1♀, 12–30.I.2015 (IBSP 197186); Cave ASF 03 (ex Abrigo PT 31) (18°59'54"S, 43°23'35"W), Am. 989, 1♀, 12–30.I.2015 (IBSP 197188), all collected by Equipe Carste et.al.; Serra do Sapo, Cave CSS 03 (ex Cave 17) (18°55'2"S, 43°25'42"W), Am. 20, 1♂, 15.XII.2010–14.I.2011 (IBSP 198501); Cave CSS 08 (ex Cave 26) (18°55'37"S, 43°25'2"W), Am. 69, 1 imm., 15.XII.2010–14.I.2011, R. Bessi et al. (IBSP 198502); Cave CSS 04 (ex Cave 18) (18°55'2"S, 43°25'42"W), Am. 194, 1♀, 15.XII.2010–14.I.2011 (IBSP 198505); Cave ASS 07 (ex Cave 32) (18°56'40"S, 43°24'24"W), Am. 82, 1♂, 15.XII.2010–14.I.2011 (IBSP 198503); Cave CSS 13 (ex Cave 33) (18°56'40"S, 43°24'23"W), Am. 93, 1♂, 15.XII.2010–14.I.2011 (IBSP 198504); Cave CSS 03 (ex Cave 17) (18°55'2"S, 43°25'42"W), Am. 320, 2♀, 15.XII.2010–14.I.2011 (IBSP 198506), all collected by R. Bessi et al.; Cave SERP_112, Am. 158, 1♀, 29.XI.2013, L.G.S. Soares leg. (IBSP 184868); Mariana, Cave FN-018 (20°12'26"S, 43°26'18"W), Am. 34, 1♀, 26.X.2012 (IBSP 189192); Cave FN, Am. 25, 1♂, 09.XII.2012 (IBSP 189190); Cave FN, Am. 29, 1♂, 17.X.2012 (IBSP 189191); Cave FN, Am. 01, 1♀ 1 imm., 29.X.2012 (IBSP 189181); Cave FN-017 (20°12'23"S, 43°26'16"W), Am. 02, 1♀ 5 imm., 31.X.2012 (IBSP 189182), all collected by BioEspeleo Cons. Amb. leg.; Barão de Cocais, Cave RF-032 (19°55'42"S, 43°30'29"W), Am. 32, 1♀, 21.III.2009, R. Andrade et al. (IBSP 196268); Cave RF-059 (19°56'3"S, 43°31'58"W), Am. 33, 1 imm., 10–21.VII.2009, (IBSP 196272); Cave RF-025 (19°55'27"S, 43°29'58"W), Am. 26, 1♀, 10–21.VII.2009 (IBSP 196273); Cave RF-058 (19°56'5"S, 43°31'32"W), Am. 34, 2♀, 10–21.VII.2009 (IBSP 196275); Cave RF-027 (19°55'28"S, 43°29'57"W), Am. 24, 1♀, 10–21.VII.2009 (IBSP 196276); Cave RF-032 (19°55'42"S, 43°30'29"W), Am. 31, 4♀ 3 imm., 10–21.III.2009 (IBSP 196278); Cave RF-049 (19°55'46"S, 43°30'47"W), Am. 27, 5♀ 3 imm., 10–21.III.2009 (IBSP 196282); Cave RF-042 (19°55'43"S, 43°30'34"W), Am.38, 1♀ 3 imm., 10–21.III.2009 (IBSP 196283; MEV; pictures), all collected by R. Andrade et al.; Cave RF-043 (19°55'43"S, 43°30'34"W), Am. 86, 1 imm., 10–21.III.2009, (IBSP 196270); Cave RF-009 (19°55'8"S, 43°29'14"W), Am. 514, 2♀, 22.VI.2009 (IBSP 196269); Cave RF-049 (19°55'46"S, 43°30'47"W), Am. 519, 1♀, 22.VI.2009 (IBSP 196271); Cave RF-032 (19°55'42"S, 43°30'29"W), Am. 517, 3♀ 1 imm., 22.VI–03.VII.2009 (IBSP 196279); Cave RF-036 (19°55'42"S, 43°30'31"W), Am. 516, 2♀, 22.VI–03.VII.2009 (IBSP 196280); Cave RF-048 (19°55'46"S, 43°30'47"W), Am. 521, 2♀, 22.VI–03.VII.2009 (IBSP 196281); Cave RF-042 (19°55'43"S, 43°30'34"W), Am. 515, 1♀, 22.VI.2009 (IBSP 196274); Cave RF-045 (19°55'44"S, 43°30'34"W), Am. 518, 1♀, 22.VI–03.VII.2009 (IBSP 196284); Cave RF-069 (19°56'23"S, 43°32'4"W); Am. 875, 1♀, 09–13.XII.2009 (IBSP 182821); Cave RF-069 (19°56'23"S, 43°32'4"W), Am. 781, 2♀, 20–25.VIII.2009 (IBSP 182818); Cave RF-080 (19°56'25"S, 43°32'39"W), Am. 791, 2♀, 20–25.VIII.2009 (IBSP 182819); Cave RF-063 (19°56'16"S, 43°32'1"W), Am. 913, 1♀, 09–13.XII.2009 (IBSP 182823), all collected by R. Bessi et al.; Mina do Baú, Cave_011 (19°57'22"S, 43°37'42"W), Am. 03, 2♂, 28.X.2016, F. Bondezan leg. (IBSP 198500); Moeda, Cave VL-37 (20°17'56"S, 43°56'46"W), Am. 84, 3♀, 29.III–03.IV.2012, J. Mascarenhas leg. (IBSP 180709); Itabirito, Cave VL-17 (20°20'30"S, 43°56'10"W), Am. 02, 1♂ 1♀, 4–25.X.2012 (IBSP 180937); Cave VL-48 (20°17'52"S, 43°56'35"W), Am. 62, 1♀, 3–6.X.2011 (IBSP 180707); Cave VL-10 (20°17'7"S, 43°56'43"W), Am. 101, 3♀, 29.III–03.IV.2012 (IBSP 180710); Cave VL-50 (20°17'56"S, 43°56'27"W), Am. 69, 1♀, 29.III–03.IV.2012 (IBSP 180708); Cave VL-48 (20°17'52"S, 43°56'35"W), Am. 59, 1♀, 3–6.X.2011 (IBSP 180938); Cave VL-48 (20°17'52"S, 43°56'35"W), Am. 60, 2♀, 3–6.X.2011 (IBSP 180706); Cave VL-52 (20°17'57"S, 43°56'27"W); Am. 11, 1♀, 3–6.X.2011 (IBSP 180705); Cave VL-51 (20°17'56"S, 43°56'27"W), Am. 5, 1♀, 3–6.X.2011 (IBSP 180704), Cave VL-25 (20°20'9"S, 43°56'5"W), Am. 104, 2♀, 07–10.V.2013 (IBSP 180853); Cave VL-25 (20°20'9"S, 43°56'5"W), Am. 105, 1♀, 07–10.V.2013 (IBSP 180854), all collected by J. Mascarenhas leg.; Cave CAP2_0020 (20°8'55"S, 43°39'22"W), Am. 1409, 1♀, 15–31.III.2016 (IBSP 198525); Cave CAP2_0020 (20°8'55"S, 43°39'22"W), Am. 1645, 1♀, 14.VII–18.IX.2016 (IBSP 198530), all collected by Equipe Carste leg.; Santa Bárbara, Serra do Gandarela, Cave GAND_0038 (20°2'30"S, 43°39'9"W), Am. 1831, 1♂, 14.VII–18.IX.2016 (IBSP 198531); Cave SG-029 (ex Cave GAND_0003) (20°6'14"S, 43°38'12"W), Am. 745, 1♀, 10.II–20.III.2014 (IBSP 198507); Cave GAND_0095 (20°3'56"S, 43°40'12"W), Am. 1011, 1♀, 10.II–20.III.2014 (IBSP 198509); Cave GAND_0033 (20°5'24"S, 43°39'9"W), Am. 1800, 1♀, 14.VII–18.IX.2016 (IBSP 198510); Cave GAND 0065 (20°4'22"S, 43°39'31"W), Am. 1898, 1♂, 15–31.III.2016 (IBSP 198516); Cave GAND_0037 (20°2'29"S, 43°39'8"W), Am. 2221, 1♀, 15–31.III.2016 (IBSP 198517); Cave GAND_0116 (20°4'11"S, 43°40'10"W), Am. 1510, 1♀, 15–31.III.2016 (IBSP 198518); Cave SG-029 (ex Cave GAND_0003) (20°6'14"S, 43°38'12"W), Am. 746, 1♀, 10.II–20.III.2014 (IBSP 198512); Cave SG-048 (20°5'9"S, 43°40'25"W), Am. 1133, 1♀, 10.II–20.III.2014 (IBSP 198513); Cave GAND_0051 (20°2'55"S, 43°40'9"W), Am. 1151, 2♀, 10.II–20.III.2014 (IBSP 198520); Cave GAND_0033 (20°5'24"S, 43°39'9"W), Am. 1801, 1♀, 14.VII–18.IX.2016 (IBSP 198521); Cave GAND_0013 (20°5'43"S, 43°40'14"W), Am. 787, 1♀, 10.II–20.III.2014 (IBSP 198523); Cave SG-043 (20°5'48"S, 43°40'28"W), Am. 2240, 1♀, 14.VII–18.IX.2016 (IBSP 198524); Cave GAND_0116 (20°4'11"S, 43°40'10"W), Am. 2111, 1♀, 14.VII–18.IX.2016 (IBSP 198526); Cave GAND_0114 (20°4'6"S, 43°40'14"W), Am. 1275, 1♀, 10.II–20.III.2014 (IBSP 198528); Cave GAND_0063 (20°4'29"S, 43°39'26"W), Am. 1298, 1♀, 15–31.III.2016 (IBSP 198529); Cave GAND_0116 (20°4'11"S, 43°40'10"W), Am. 2110, 1♀, 14.VII–18.IX.2016 (IBSP 198532), all collected by Equipe Carste leg.; Rio Acima, Serra do Gandarela, Cave SG-013 (ex Cave GAND_0015) (20°5'39"S, 43°41'6"W), Am. 794, 4♀, 10.II–20.III.2014 (IBSP 198522); Cave SG-016 (20°5'40"S, 43°40'51"W), Am. 2199, 1♂, 14.VII–18.IX.2016 (IBSP 198511); Cave SG-051 (20°5'50"S, 43°40'43"W), Am. 1156, 1♀, 10.II–20.III.2014 (IBSP 198514); Cave SG-051 (20°5'50"S, 43°40'43"W), Am. 1154, 1♀ 2 imm., 10.II–20.III.2014 (IBSP 198515); Cave GAND_82 (20°5'40"S, 43°40'49"W), Am. 1302, 1♀, 15–31.III.2016 (IBSP 198508); Cave SG-012 (ex Cave GAND_0102) (20°5'38"S, 43°41'5"W), Am. 1017, 1♀, 10.II–20.III.2014 (IBSP 198519); Cave SG-012 (ex Cave GAND_0102) (20°5'38"S, 43°41'5"W), Am. 2039, 2♀, 14.VII–18.IX.2016 (IBSP 198527), all collected by Equipe Carste leg.

###### Etymology.

The specific epithet is a noun taken in apposition and refers to the type locality.

###### Diagnosis.

**Figure 8.**
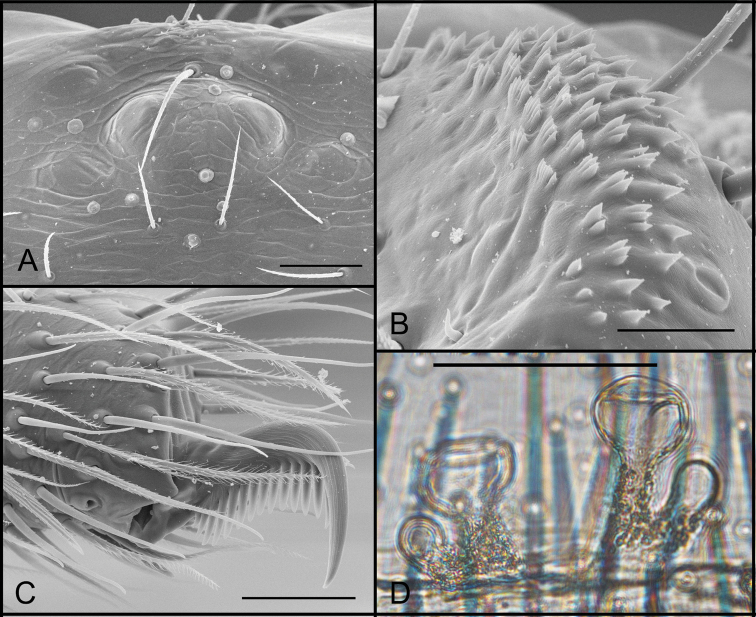
*Tontonmatodentro* sp. n., (female, Conceição do Mato Dentro, Minas Gerais, IBSP 196239), **A** eyes, dorsal view **B** serrula, lateral view **C** palpal claw, prolateral view **D** female genitalia, dorsal view. Scale bars: 50 μm (**A, B, C**); 100 μm (**D**).

Males of *Tontonmatodentro* sp. n. are similar to those of other species in the genus by the piriform bulb, differing by the apex of embolus, which is enlarged and slightly flattened (Fig. 7C–G). Females resemble *T.emboaba* by the similar internal lobe of the spermathecae, but differ by the elongated external lobe (Fig. 8D).

###### Description.

**Male (holotype).** Coloration: carapace and legs whitish, abdomen yellowish. Total length 1.95. Carapace 0.95 long, 0.65 wide. Abdomen 1.0 long. Fovea 0.13. Clypeus 0.03. Eyes: six, the lateral eyes are vestigial, PME 0.02, lateral eyes are not visible to be measured (Fig. 5A). Labium 0.1 long, 0.15 wide. Sternum 0.45 wide, 0.58 long. Serrula with 4–5 rows of teeth clumped in series (Fig. 7B). Palp: femur 0.54/ patella 0.29/ tibia 0.41/ tarsus 0.28/ total 1.52. Leg I: femur 0.73/ patella 0.39/ tibia 0.36/ metatarsus 0.34/ tarsus 0.37/ total 2.19 used on SEM pictures; II femur 0.73/ patella 0.39/ tibia 0.36/ metatarsus 0.34/ tarsus 0.37/ total 2.19; III 0.63/ 0.33/ 0.45/ 0.39/ 0.38/ 2.18; IV 0.78/ 0.39/ 0.59/ 0.59/ 0.45/ 2.8. Leg formula 4123. Spination: palp tibia p3, tarsus d5ap; leg II tibia v4-3ap, p1, metatarsus v2-2ap; III patella p1, r2, tibia d1, v4-2ap, p1, r1, metatarsus d2, v2-3ap, p2, r1; IV patella v1, p2, r2, tibia d1, v3-3ap, p3, r3, metatarsus d3, v2-3ap, p2, r1. Tibia I with three apical ventral spines. Palpal tibia two times the size of cymbium, with the bulb resting in a ventral depression. Cymbium as long as wide with a short prolateral lobe, with five apical spines (Fig. 7D, E). Bulb piriform, embolus elongated with the same size of tegulum, with strong medial curvature, and apex rounded (Fig. 7C–G). PLS basal, medial and apical segments, 0.22, 0.18, 0.16 long.

**Female (paratype).** Coloration as in male. Total length 2.18. Carapace 1.0 long, 0.74 wide. Abdomen 1.18 long. Fovea 0.07. Clypeus 0.03. Eyes: six, the lateral eyes vestigial, ALE 0.03, PME 0.01, PLE 0.07 (Fig. 8A). Labium 0.1 long, 0.16 wide. Sternum 0.57 wide, 0.58 long. Serrula as in male (Fig. 8B). Palp: femur 0.52/ patella 0.29/ tibia 0.32/ tarsus 0.33/ total 1.46. Leg I femur 0.64/ patella 0.39/ tibia 0.46/ metatarsus 0.32/ tarsus 0.29/ total 2.10; II 0.59/ 0.34/ 0.38/ 0.30/ 0.26/ 1.87; III 0.47/ 0.26/ 0.33/ 0.36/ 0.33/ 1.75; IV 0.65/ 0.36/ 0.52/ 0.46/ 0.37/ 2.36. Leg formula 4123. Spination: palp tibia v1-2ap, tarsus v2; leg I tibia v1-2ap, metatarsus v2-2ap; II patella p1, tibia v3-3ap, p3, metatarsus v2-3ap, p1; III patella p2, r1, tibia d1, v3-3ap, p1, r1, metatarsus d3, v3-3ap, p1; IV patella p1, tibia d1, v3-3ap, p2, r1, metatarsus d2, v3-3ap, p2, r2. Genitalia with internal lobe with large and rounded apex, external lobe connected to the basis with elongated duct and discrete globous apex, wide spermathecae basis, glands in both lobes, scattered irregularly (Fig. 8D, left side collapsed). PLS: basal, medial and apical segments, 0.22, 0.18, 0.16 long.

###### Natural history.

The specimens of *Tontonmatodentro* sp. n. were collected in the caves in the Quadrilátero Ferrífero Iron Region, on the floor. This species is classified as troglobite.

###### Distribution.

Morro do Pilar and Conceição de Mato Dentro, in the state of Minas Gerais (Fig. 13).

##### Tonton sapalo sp. n.

Taxon classificationAnimaliaAraneaeMicrostigmatidae

http://zoobank.org/70BB233E-191E-4C80-AFD8-3DCEC0E4CD7F

Figures 9 , 13

Masteria sp.: De Maria et al. 2009: 219 (examined, misidentification).

###### Types.

Male holotype from RPPN Mata Samuel de Paula, 20°0'04"S, 43°52'16"W, Nova Lima, Minas Gerais, Brazil, deposited in UFMG 2520; female paratype, with same data, deposited in UFMG 2521.

###### Other material examined.

BRAZIL. Minas Gerais, Nova Lima, RPPN Mata Samuel de Paula (20°0'2"S, 43°52'14"W), 2♂, no more data (UFMG 2844; MEV; photos).

###### Etymology.

The specific name is an arbitrary combination of letters.

###### Diagnosis.

**Figure 9.**
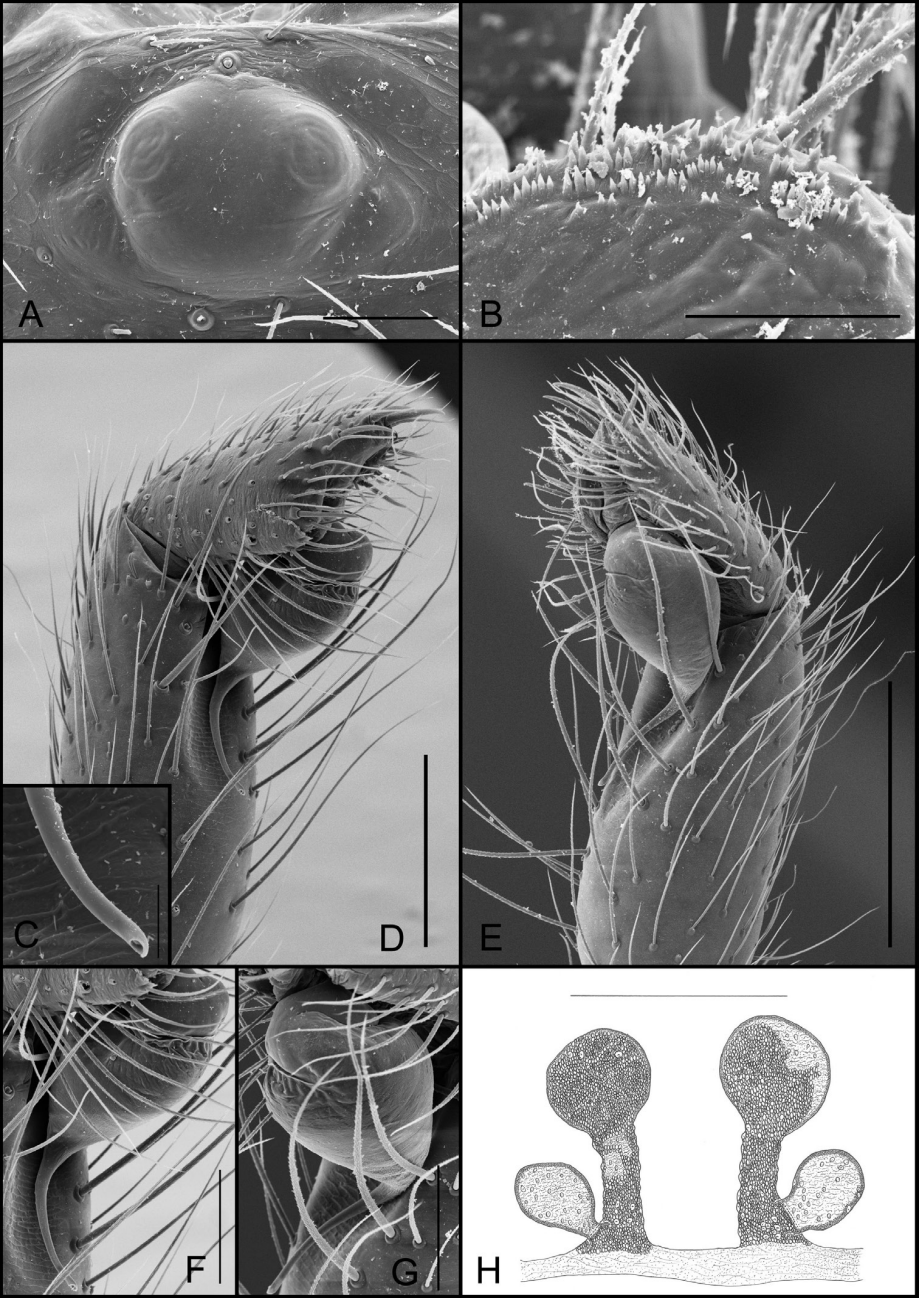
*Tontonsapalo* sp. n. (male and female, RPPN Mata Samuel de Paula, Nova Lima, Minas Gerais, UFMG 2844, male, UFMG 2521, female), **A** eyes, dorsal view **B** serrula, lateral view **C** embolous tip, ventral view **D** palp, prolateral view **E** palp, retrolateral view **F** bulb, retrolateral view, **G** bulb, prolateral view, **H** female genitalia, dorsal view. Scale bars: 50 μm (**A**); 40 μm (**B**); 20 μm (**C**); 200 μm (**D, E**); 100 μm (**F, G, H**).

Males of *Tontonsapalo* sp. n. are similar to the other species in the genus with the piriform palp bulb, but differ by a strong basal curvature on the embolus and the embolus apex slightly flat and digitiform (Fig. 9C–G). Females resemble *T.emboaba* by the similar internal lobe of the spermathecae, but differ by the narrow internal lobe of the spermathecae and longer external lobe (Fig. 9H).

###### Description.

**Male (Holotype).** Coloration uniformly yellowish. Total length 2.32. Carapace 1.14 long, 0.88 wide. Abdomen 1.18 long. Fovea 0.11. Clypeus 0.03. Eyes: six, the lateral vestigial, PME on a slightly elevated basis, ALE 0.06, PME 0.03, PLE 0.06 (Fig. 9A). Labium 0.12 long, 0.22 wide. Sternum 0.56 wide, 0.70 long. Serrula in 3–4 rows of the teeth clumped in series. Palp: femur 0.65/ patella 0.34/ tibia 0.59/ tarsus 0.34/ total 1.92. Leg I femur 0.85/ patella 0.50/ tibia 0.62/ metatarsus 0.49/ tarsus 0.37/ total 2.83; II 0.75/ 0.41/ 0.46/ 0.39/ 0.34/ 2.35; III 0.67/ 0.36/ 0.46/ 0.46/ 0.41/ 2.36; IV 0.85/ 0.46/ 0.65/ 0.69/ 0.49/ 3.14. Leg formula 4132. Spination: palp tibia p2, tarsus d5ap; leg I tibia v3-3ap, metatarsus v3ap; II tibia v3-2ap, p1, metatarsus v1-3ap, p1; III patella p2, r1, tibia d1, v3-3ap, p2, r1, metatarsus d1, v1-3ap, p1, r2; IV patella p1, r1, tibia d1, v2-3ap, p2, r3, metatarsus d2, v1-3ap, p2, r2. Tibia I with three apical ventral spines. Palpal tibia two times the size of cymbium, with the bulb resting in a ventral depression. Cymbium as long as wide with a short prolateral lobe, with five apical spines (Fig. 9C–E). Bulb piriform, embolus as long as the tegulum (Fig. 9C–G). PLS basal, medial and apical segments 0.34, 0.24, 0.2 long.

**Female (Paratype).** Coloration as in male. Total length 3.04. Carapace 1.46 long, 1.1 wide. Abdomen 1.58 long. Fovea 0.12. Clypeus 0.04. Eyes as in male, ALE 0.07, PME 0.05, PLE 0.08. Labium 0.14 long, 0.18 wide. Sternum 0.7 wide, 0.8 long. Serrula as in male. Palp: femur 0.78/ patella 0.38/ tibia 0.52/ tarsus 0.52/ total 2.2. Leg I femur 0.94/ patella 0.59/ tibia 0.65/ metatarsus 0.49/ tarsus 0.41/ total 3.08; II 0.85/ 0.55/ 0.59/ 0.46/ 0.42/ 2.87; III 0.78/ 0.49/ 0.55/ 0.55/ 0.49/ 2.86; IV 1.04/ 0.56/ 0.72/ 0.72/ 0.52/ 3.56. Leg formula 4123. Spination: palp tibia v4-1ap, tarsus v2; leg I patella p1, tibia v3-2ap, p1, metatarsus v3-2ap, p1; II patella p2, tibia v3-3ap, p2, metatarsus v3-2ap, p1; III patella d2, v1, p2, tibia d2, v1-3ap, p2, r2, metatarsus d2, v2-3ap, p1, r2; IV patella d1, v1, p1, r1, tibia d1, v2-3ap, p2, r2, metatarsus d4, v2-3ap, p1, r1. Genitalia with internal lobe long with globous apex, external lobe connected to a basis and globous apex, glands in both lobes, scattered irregularly (Fig. 9H). PLS: basal, medial and apical (lost) segments, 0.46, 0.16 long.

###### Natural history.

The specimens were collected in the litter with pitfall traps.

###### Distribution.

Known only from the type locality (Fig. 13).

##### Tonton ipiau sp. n.

Taxon classificationAnimaliaAraneaeMicrostigmatidae

http://zoobank.org/6840274C-AFD2-4926-BE30-ACB968A49098

Figures 10 , 14

###### Types.

Male holotype and female paratype from Ipiaú, (14°8'20"S, 39°44'52"W), Bahia, Brazil, I.2007, C. Máximo leg., deposited in IBSP 14522 and IBSP 14520, respectively.

###### Other material examined.

BRAZIL. **Sergipe**: Itabaiana, Estação Ecológica da Serra de Itabaiana (10°40'S, 37°25'W), 1♂, 14–20.IX.1999, A.D. Brescovit et al. (IBSP 13206; MEV); 1♀, 14–20.IX.1999 (IBSP 13217); 1♂, 14–20.IX.1999 (IBSP 13207); 1♂, 14–20.IX.1999 (IBSP 13205); Santa Luzia do Itanhy, Mata do Crasto (11°21'7"S, 37°26'57"W), 1♂, 9–13.IX.1999 (IBSP 198560), all collected by A.D. Brescovit et al.; **Bahia**: Ipiaú (14°7'58"S, 39°45'10"W), 1♀, I.2007, C. Máximo leg. (IBSP 14521; MEV); 1♂, I.2007, C. Máximo leg. (IBSP 14523; MEV); Lafaiete Coutinho (13°39'23"S, 40°13'17"W), 1♂, VII.2006–VII.2007, J. Romão leg. (IBSP 14734); 1♀, VII.2006–VII.2007, J. Romão leg. (IBSP 14741); Wenceslau Guimarães (13°38'39"S, 39°45'36"W), pitfall trap QD53, 1♂, 14–21.I.2011, C. Leite leg. (IBSP 198556); Wenceslau Guimarães (13°38'5"S, 39°40'6"W), pitfall trap QD47, 2♀, 14–21.I.2011 (IBSP 198557); pitfall trap QD 43, 1♂, 14–21.I.2011 (IBSP 198558); pitfall trap QD 17, 1♀, 14–21.I.2011 (IBSP 198559), all collected by C. Leite leg.; Jaguaripe (13°6'53"S, 38°54'6"W), pitfall trap QD98, 2♂, 18.II–02.III.2011 (IBSP 198551); pitfall trap QD89, 1♀, 18.II–02.III.2011 (IBSP 198552); pitfall trap QD55, 2♂, 18.II–02.III.2011 (IBSP 198553); pitfall trap QD89, 1♂, 18.II–02.III.2011 (IBSP 198554); pitfall trap QD55, 1♀, 18.II–02.III.2011 (IBSP 198555), all collected by C. Leite.

###### Etymology.

The specific epithet is a noun taken in apposition and refers to the type locality.

###### Diagnosis.

**Figure 10.**
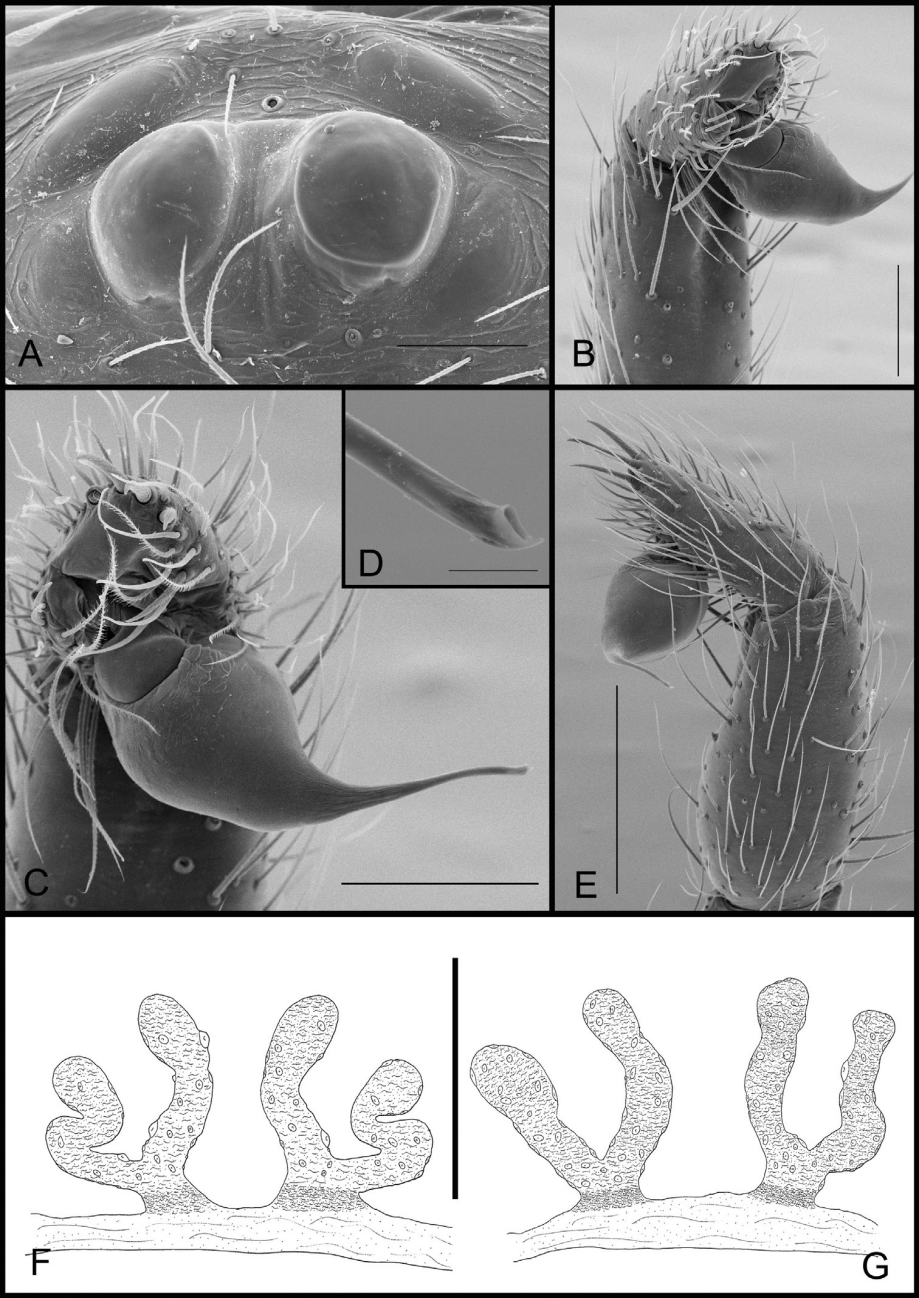
*Tontonipiau* sp. n., (male, Ipiaú, Bahia, IBSP 14523), **A** Eyes, dorsal view **B** palp, prolateral view **C** bulb, ventral view **D** embolous tip **E** palp, retrolateral view **F, G** variation of female genitalia. Scale bars: 50 μm (**A**); 100 μm (**B, C**); 10 μm (**D**); 200 μm (**E**); 100 μm (**F, G**).

Males of *Tontonipiau* sp. n. resemble the males of other species with piriform palpal bulb, but differ by the embolus basis wide and tapering to the apex (Fig. 10B–E). Females differ from the other species by spermathecae with long and narrowed lobes with similar size (Fig. 10 F-G).

###### Description.

**Male** (Holotype). Coloration uniformly yellowish. Total length 1.8. Carapace 0.84 long, 0.66 wide. Abdomen 0.96 long. Fovea 0.11. Clypeus 0.02. Eyes: six, the lateral eyes are vestigial, PME largest in proportion to the other eyes, ALE 0.06, PME 0.08, PLE 0.05 (Fig. 10A). Labium 0.06 long, 0.16 wide. Sternum 0.44 wide, 0.5 long. Serrula as in *T.sapalo* sp. n. Palp: femur 0.43/ patella 0.26/ tibia 0.33/ tarsus 0.24/ total 1.26. Leg I: femur 0.67/ patella 0.33/ tibia 0.43/ metatarsus 0.38/ tarsus 0.34/ total 2.15; II 0.58/ 0.34/ 0.37/ 0.33/ 0.32/ 1.94; III 0.51/ 0.26/ 0.37/ 0.39/ 0.33/ 1.86; IV 0.72/ 0.35/ 0.54/ 0.52/ 0.39/ 2.52. Leg formula 4123. Spination: palp tibia v3, tarsus d5ap; leg I: tibia v1-3ap, metatarsus v3-3ap; II: patella v1, p1, tibia v2-3ap, p2, r2, metatarsus v3-2ap, p2; III: patella p2, r1, tibia d2, v2-2ap, p2, r1, metatarsus d2, v2-3ap, p3, r1; IV: patella v2, p1, tibia d2, v2-2ap, p2, r2, metatarsus d2, v3-3ap, p2, r1. Tibia I with three apical ventral spines. Palpal tibia with two times the size of cymbium, with the bulb resting in a ventral depression. Cymbium as long as wide with a short prolateral lobe, with five apical spines (Fig. 10B, E). Bulb piriform, tegulum tapering to the embolus, embolus as long as the tegulum, with light medial curvature, apex of embolus rounded (Fig. 10B–E). PLS basal, medial and apical segments 0.32, 0.24, 0.21 long.

**Female (Paratype).** Coloration as in male. Total length 2.22. Carapace 1.06 long, 0.9 wide. Abdomen 1.16 long. Fovea 0.08. Clypeus 0.06. Eyes as in male, ALE 0.08, PME 0.05, PLE 0.03. Labium 0.1 long, 0.24 wide. Sternum 0.6 wide, 0.64 long. Serrula as in male. Palp: femur 0.59/ patella 0.37/ tibia 0.43/ tarsus 0.46/ total 1.85. Leg I: femur 0.99/ patella 0.52/ tibia 0.65/ metatarsus 0.50/ tarsus 0.46/ total 3.12; II 0.83/ 0.47/ 0.46/ 0.42/ 0.37/ 2.55; III 0.72/ 0.41/ 0.43/ 0.46/ 0.37/ 2.39; IV 0.91/ 0.46/ 0.69/ 0.59/ 0.46/ 3.11. Leg formula 1423. Spination: palp patella v1, tibia v4-2ap, tarsus v7; leg I: patella p1, tibia v3-2ap, p1, metatarsus v5-3ap, p1; II: patella p2, tibia v3-2ap, p2, metatarsus v3-3ap, p2; III: patella v1, p2, r1, tibia d2, v3-3ap, p2, r1, metatarsus d2, v2-3ap, p2, r2; IV: patella p1, r1, tibia d3, v2-3ap, p1, r2, metatarsus d2, v1-3ap, p1, r2. Genitalia with spermathecae sharing the basis, slightly projected, internal lobe little longer than the external lobe, glands in both lobes, scattered irregularly (Fig. 10G-H). PLS: basal, medial and apical segments, 0.4, 0.2, 0.3 long.

**Variation**. Some females can be have the external lobe slightly sinuous (Fig. 10F).

###### Natural history.

All specimens were collected in the litter with pitfall traps.

###### Distribution.

Atlantic forest, in the states of Sergipe and Bahia (Fig. 13).

##### Tonton quiteria sp. n.

Taxon classificationAnimaliaAraneaeMicrostigmatidae

http://zoobank.org/86A25CD4-E49D-49DE-937F-486D5BAD86F5

Figures 11 , 14

###### Types.

Holotype male and paratype female from Santa Quitéria do Maranhão (3°30'57"S, 42°32'49"W), Maranhão, Brazil, XII/1999, M. Mendonça leg., deposited in IBSP 198545 and 198546.

###### Other material examined.

BRAZIL. Maranhão: Santa Quitéria do Maranhão (3°29'49"S, 42°34'16"W), 1♂, XII.1999 (IBSP 198547); 1♂, XII.1999 (IBSP 198548); 1♂, XII.1999 (IBSP 198549); 1♂, XII.1999 (IBSP 198550; MEV); all collected by M. Mendonça.

###### Etymology.

The specific epithet is a noun taken in apposition and refers to the type locality.

###### Diagnosis.

**Figure 11.**
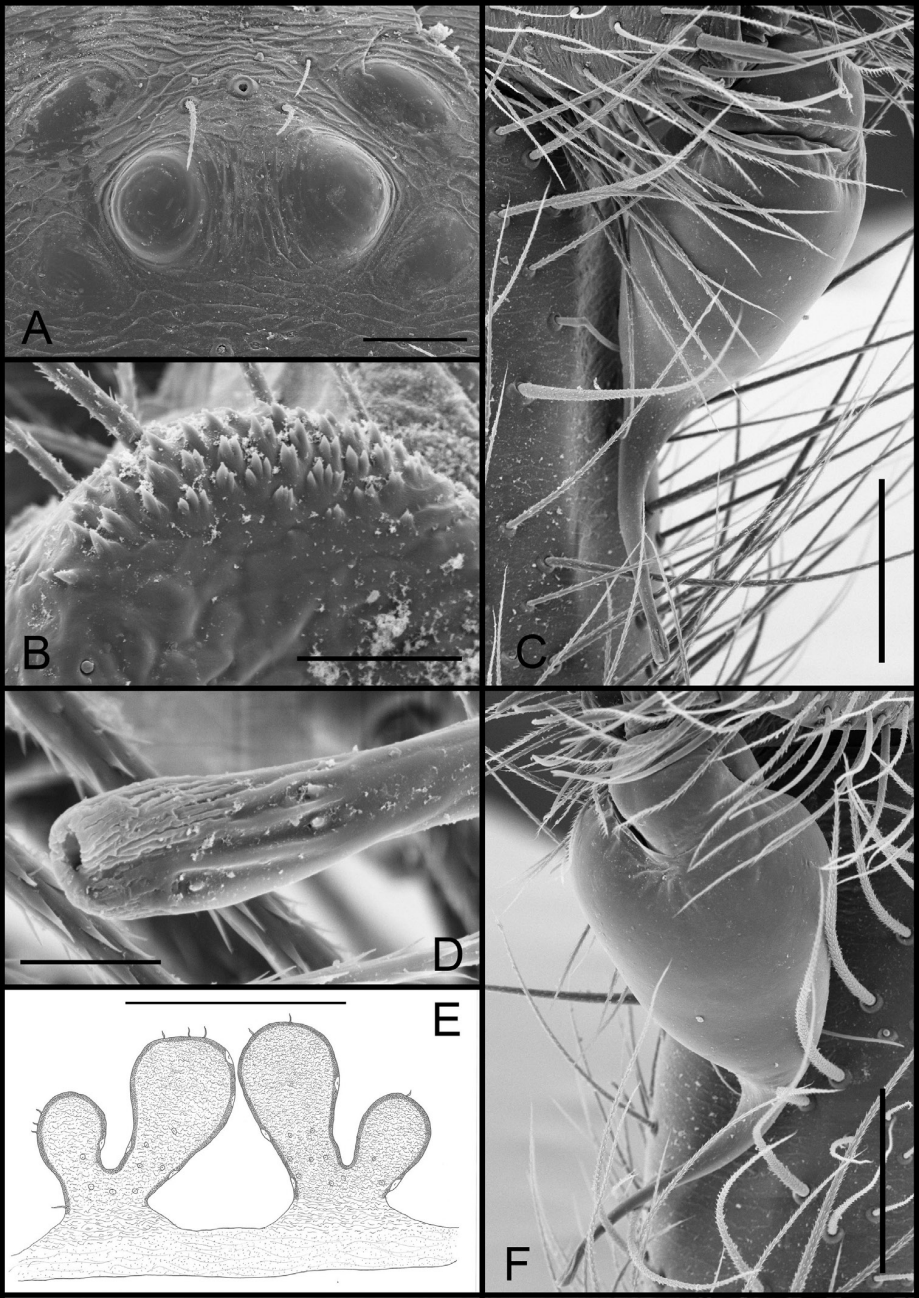
*Tontonquiteria* sp. n., (male and female, Santa Quitéria do Maranhão, Maranhão, IBSP 198550, male, IBSP 198545, female), **A** eyes, dorsal view **B** serrula, lateral view **C** bulb, prolateral view **D** embolous tip **E** female genitalia **F** bulb, retrolateral view. Scale bars: 50 μm (**A**); 30 μm (**B**); 100 μm (**C**); 10 μm (**D**); 100 μm (**E, F**).

Males of *Tontonquiteria* sp. n. resemble those of other species by the piriform palpal bulb, but differ by the embolus basis wide and tapering to the apex (Fig. 11C); they are close to those of *T.ipiau* sp. n. by the wide basis of embolus (Fig. 10C), differing by the embolus apex truncate with corrugations (Fig. 11D). Females differ from the other species by the spermathecae with short and thickened lobes (Fig. 11E).

###### Description.

**Male (holotype).** Coloration: Carapace in dorsal and ventral view, and legs yellowish, abdomen yellowish. Total length 2.26. Carapace 1.10 long, 0.82 wide. Abdomen 1.16 long. Fovea 0.12. Clypeus 0.04. Eyes: six, the lateral eyes are vestigial, AME absent, ALE 0.05, PME 0.05, PLE 0.04 (Fig. 11A). Labium 0.08 long, 0.18 wide. Sternum 0.54 wide, 0.70 long. Serrula with teeth clumped in series of the several rows. Palp: femur 0.62/ patella 0.30/ tibia 0.50/ tarsus 0.30/ total 1.72; Leg I: femur 0.85/ patella 0.46/ tibia 0.58/ metatarsus 0.46/ tarsus 0.45/ total 2.80; II: 0.76/ 0.39/ 0.52/ 0.39/ 0.38/ 2.44; III: 0.67/ 0.37/ 0.45/ 0.46/ 0.46/ 2.41; IV: 0.82/ 0.45/ 0.64/ 0.62/ 0.47/ 3.00; Leg formula 4123. Spination: palp tibia v2, tarsus d6ap; leg I patella p1, tibia v5-2ap, p2, metatarsus v2-2ap, p1; II patella v2, p1, tibia v3-3ap, p2, metatarsus v1-3ap, p2; III patella v1, p1, r1, tibia d4, v2-2ap, p2, r2, metatarsus d4, v2-3ap, p1, r1; IV patella v1, r1, tibia v3-3ap, p2, r1, metatarsus d3, v2-3ap, p2, r2. Tibia I with two apical ventral spines. Palpal tibia two or three times the size of cymbium, with the bulb resting in a ventral depression. Cymbium as long as wide with a short prolateral lobe, with six apical spines. Piriform palpal bulb, tegulum basis wide tapering to the embolus, embolus elongated with the same size of tegulum, with light medial curvature, apex of embolus truncate with three or four corrugated grooves (Fig. 11B-D, F). PLS basal, medial and apical segments 0.37, 0.26, 0.22 long.

**Female (paratype).** Coloration: Same as in male. Total length 2.76. Carapace 1.28 long, 0.96 wide. Abdomen 1.48 long. Fovea 0.16. Clypeus 0.04. Eyes: six, the lateral eyes are vestigial, AME absent, ALE 0.08, PME 0.06, PLE 0.07. Labium 0.10 long, 0.22 wide. Sternum 0.62 wide, 0.78 long. Serrula with teeth clumped in series of the several rows. Palp: femur 0.60/ patella 0.30/ tibia 0.26/ tarsus 0.32/ total 1.48; Leg I: femur 0.86/ patella 0.55/ tibia 0.55/ metatarsus 0.41/ tarsus 0.37/ total 2.74; II: 0.78/ 0.46/ 0.43/ 0.39/ 0.36/ 2.42; III: 0.68/ 0.34/ 0.39/ 0.37/ 0.39/ 2.17; IV: 0.81/ 0.43/ 0.59/ 0.52/ 0.49/ 2.84; Leg formula 4123. Spination: palp tibia v2-3ap, tarsus v4; leg I patella v2, p1, tibia v2-3ap, p2, metatarsus v2-2ap; II patella v1, tibia v2-2ap, p2, metatarsus v1-3ap, p2; III patella v1, p2, r1, tibia d2, v3-3ap, p2, r2, metatarsus d2, v2-3ap, p2, r2; IV patella r1, tibia v3-2ap, p2, r2, metatarsus d4, v3-3ap, p1, r2. Genitalia with two spermathecae, bilobed, sharing a basis slightly projected, internal lobe with duct short and thickened, longer than the external lobe, external lobe short and thickened, glands in both lobes, scattered irregularly (Fig. 11E). PLS: basal, medial and apical segments, 0.36, 0.24, 0.20 long.

###### Distribution.

Known only from the type locality, Santa Quitéria do Maranhão in the state of Maranhão (Fig. 13).

##### Tonton emboaba

Taxon classificationAnimaliaAraneaeMicrostigmatidae

(Pedroso, Baptista & Bertani) comb. n.

Figures 12 , 13

Masteria emboaba Pedroso, Baptista & Bertani, 2015: 60 (Holotype male from Serra da Gandarela, Caeté (20°01'40"S, 43°40'52"W, 1484 m a.s.l.), Minas Gerais, Brazil, May 2011, M.E. Bichuette leg., deposited in MNRJ 4540 (not found, not examined). Paratypes: two females and one immature from same locality and data, deposited in MNRJ 4540); two females and ten immature from Caeté, next to cave AP_09 (20°01'33"S, 43°40'54"W), 1439 m a.s.l., 09.VII.2011, Equipe Aracno leg., deposited in MNRJ 4388; one immature from Santa Bárbara, next to cave AP_31 (20°02'14"S, 43°40'38"W, 1443 m a.s.l., 08.VII.2011, Equipe Aracno leg., deposited in MNRJ 4380 (not found, not examined).

###### Other material examined.

BRAZIL. Minas Gerais, Caeté, Cave AP-10 (20°1'34"S, 43°40'56"W), Am. 380, 1♀, 14–21.XI.2008 (IBSP 196207); Cave AP-10 (20°1'34"S, 43°40'56"W), Am. 145, 6♀, 19–23.VII.2008 (IBSP 196208); Cave AP-22 (20°2'10"S, 43°41'19"W), Am. 152, 1♀, 19–23.VII.2008 (IBSP 196209); Cave AP-22 (20°2'10"S, 43°41'19"W), Am. 311, 1♀, 14–21.XI.2008 (IBSP 196210); Cave AP-15 (20°1'41"S, 43°40'53"W), Am. 197, 2♀, 19–23.VII.2008 (IBSP 196211); Cave AP-12 (20°1'32"S, 43°40'53"W), Am. 463, 1♂ 1♀, VII.2008 (IBSP 196212; detached epigynum); Cave AP-16 (20°1'41"S, 43°40'53"W), Am. 183, 1♀, 19–23.VII.2008 (IBSP 196213); Cave AP-09 (20°1'34"S, 43°40'55"W), Am. 104, 1♀, 19–23.VII.2008 (IBSP 196214); Cave AP-01 (20°0'47"S, 43°40'25"W), Am. 131, 2♀, 19–23.VII.2008 (IBSP 196215); Cave AP-39 (20°2'15"S, 43°40'39"W); Am. 63, 1♀, 19–23.VII.2008 (IBSP 196216; detached epigynum); Cave AP-13 (20°1'41"S, 43°40'52"W), Am. 96, 1♀, 19–23.VII.2008 (IBSP 196217; detached epigynum); Cave AP-16 (20°1'41"S, 43°40'53"W), Am. 18, 1♂, 19–23.VII.2008 (IBSP 196218; MEV); Cave AP-16 (20°1'41"S, 43°40'53"W), Am. 631, 1♀, 19–23.VII.2008 (IBSP 196223); Cave AP-16 (20°1'41"S, 43°40'53"W), Am. 395, 1♀, 19–23.VII.2008 (IBSP 196225); Cave AP-47 (20°2'12"S, 43°41'32"W), Am. 44, 1♀ 2imm., 12–21.XI.2009 (IBSP 196221); Cave AP-47 (20°2'12"S, 43°41'32"W), Am. 47, 1♀ 2 imm., 12–21.XI.2009 (IBSP 196228); Cave AP-54 (20°1'41"S, 43°40'53"W), Am. 06, 6♀ 3 imm., 12–21.XI.2009 (IBSP 196229); Cave AP-47 (20°2'12"S, 43°41'32"W), Am. 210, 6♀ 2 imm., 13–17.IV.2010 (IBSP 196230); Cave AP-58 (20°1'35"S, 43°41'9"W), Am. 162, 1♀, 12–21.XI.2009, (IBSP 196233); Cave AP-33 (20°2'16"S, 43°40'39"W), Am. 300, 1♂ 1♀, 14–21.XI.2008 (IBSP 196219; MEV), all collected by R. Bessi et al.; Cave AP-25 (20°2'43"S, 43°40'57"W), Am. 652, 1♀, 02–07.I.2012, Karina leg. (IBSP 196222); Cave AP-28 (20°2'45"S, 43°40'58"W), Am. 443, 1♀, 30.VI–15.VII.2011, Karina leg. (IBSP 196226); Cave AP-08 (20°1'35"S, 43°40'47"W), Am. 604, 1♂, 12–15.XII.2011, Juliana leg. (IBSP 196224); Cave AP-12 (20°1'32"S, 43°40'53"W), Am. 488, 1 imm., 30.VI–15.VII.2011, Luiza leg. (IBSP 196231); Cave AP-18 (20°1'44"S, 43°41'0"W), Am. 405, 1♀, 30.VI–15.VII.2011, Luiza leg. (IBSP 196232); Cave AVG 48 (19°49'20"S, 43°41'43"W), Am. 5, 3♀, 02.IX.2014, M.P.A. Oliveira leg. (IBSP 198497); Santa Bárbara, Cave AP-25 (20°2'43"S, 43°40'57"W), Am. 438, 1♀, 30.VI–08.VII.2011, R. Bessi et al. leg. (IBSP 198499); Rio Acima, Cave VG-01 (20°9'9"S, 43°49'0"W), Am. 154, 5♀, 2–10.VIII.2011, I. Cizauskas leg. (IBSP 198498); Cave AP-67 (20°0'54"S, 43°40'36"W), Am. 23, 2 imm., 12–21.XI.2009, R. Bessi leg. (IBSP 196206).

###### Diagnosis.

**Figure 12.**
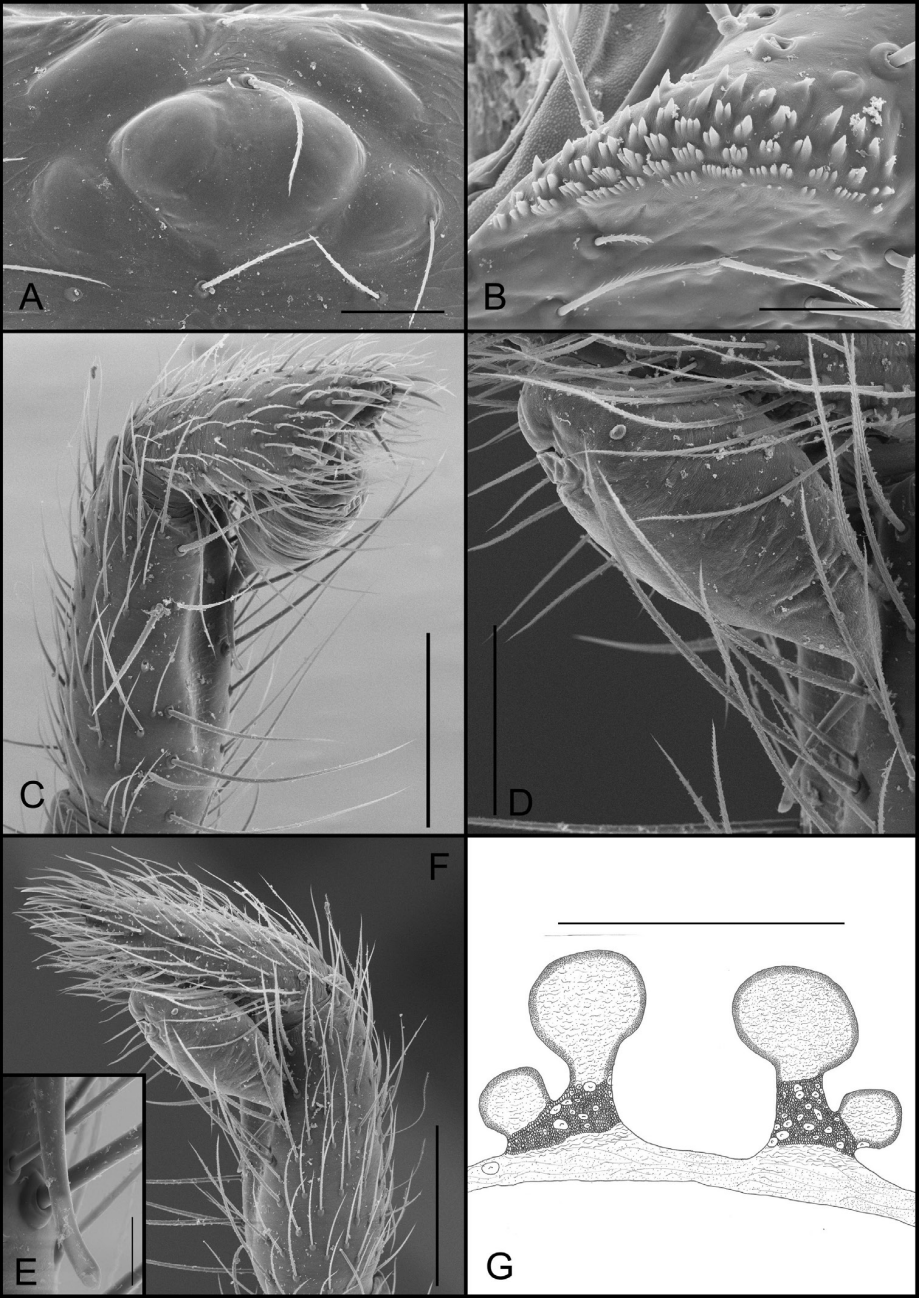
*Tontonemboaba* (male IBSP 196218 and female IBSP 169216, Caeté, Minas Gerais), **A** eyes, dorsal view **B** serrula, lateral view **C** palp, prolateral view **D** bulb, retrolateral view **E** embolous tip **F** palp, retrolateral view **G** female genitalia. Scale bar: 50 μm (**A**); 30 μm (**B**); 200 μm (**C**); 100 μm (**D**); 20 μm (**E**), 200 μm (**F**), 100 μm (**G**).

Males of *T.emboaba* have only two small spines on apex of tibia I and embolus with a curvature on basis (Fig. 12 B-D); they are close to males in other species of the genus by the piriform bulb, but can be easily distinguished by the curvature on embolus basis (Fig. 12B-D). Females present the spermathecae with the external lobes small in relation to the internal lobes (Fig. 12G), and are related to those of *T.matodentro* by the similar internal lobe of the spermathecae, but differ by the short external lobe (Fig. 12G).

###### Description.

Male and female described by Pedroso et al. 2015: 60, figs 1–16.

New data: male with six eyes, the lateral eyes are vestigial, AME absent. ALE 0.02, PME 0.04, PLE 0.02 (Fig. 12A). Serrula with 4–5 rows of clumped teeth in series. Palp with excavated distally tibia, tegulum piriform and embolus with half of length of tegulum (Figs 12C–F). Female with eyes as in male, ALE 0.02, PME 0.05, PLE 0.02. Serrula as in male. Genitalia with bilobed spermathecae, with projected internal lobe, with elongated duct, longer than the external lobe; external lobe rounded; glands in both lobes, scattered irregularly and narrow basis of genitalia (Fig. 12G).

###### Remarks.

In the original description from Pedroso et al. (2015) this species was included in Dipluridae, in the genus *Masteria*. After analyzing a large amount of material from the type locality, we conclude that the species belongs to Microstigmatidae: Micromygalinae by having serrula arranged in teeth clumped in a series of rows (Fig. 12B), and an integument with scales. With MEV photography we also observed the presence of six eyes and not two, as originally described, with the lateral eyes being almost vestigial, and the posterior median clearly visible (Fig. 12A).

###### Distribution.

**Figure 13.**
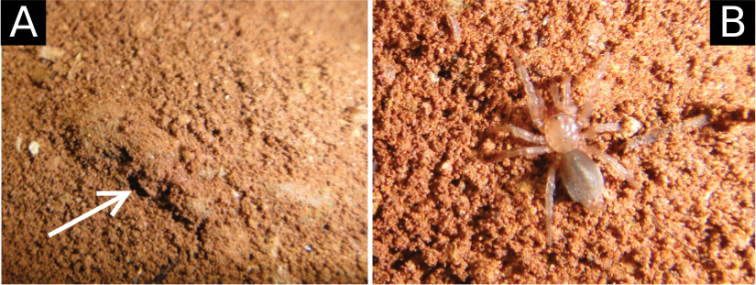
*Tontonitabirito* sp. n. **A** silk shelter with single opening **B** Female, dorsal view, from Cave VG-01, Itabirito, Minas Gerais, Brazil.

Only known from the caves on canga areas of the Caeté and Santa Bárbara, in the state of Minas Gerais (Fig. 13).

**Figure 14.**
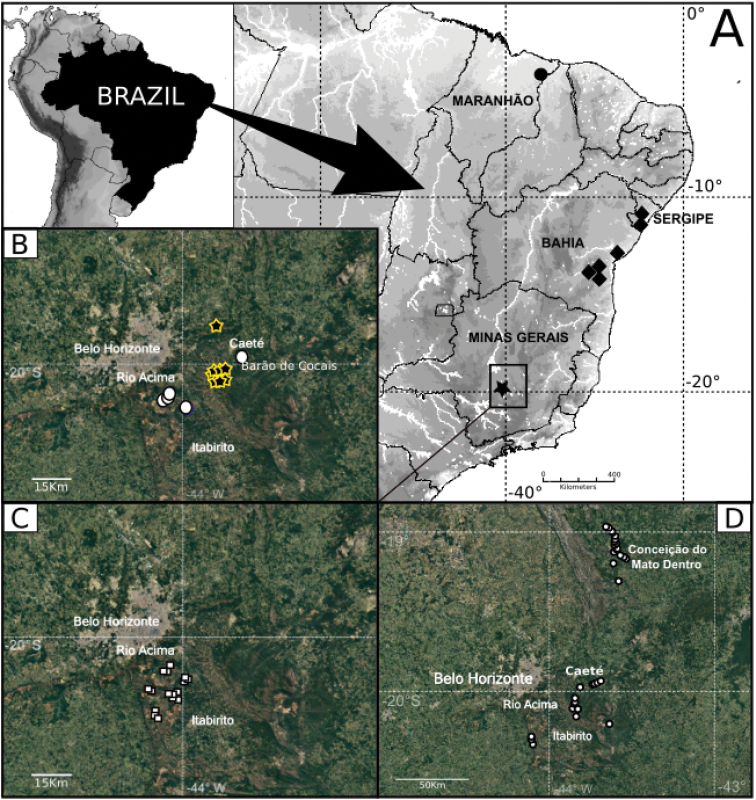
Distribution maps. Epigean fauna. **A***T.sapalo* sp. n., green star, *T.ipiau* sp. n., orange losangue and *T.quiteria* sp. n., yellow circles. Caverniculous fauna **B***T.emboaba* red star and *T.itabirito* sp. n., blue pentagon **C***T.queca* sp. n., red square **D***T.matodentro* sp. n., red circles.

## Discussion

The family Microstigmatidae is currently divided into two subfamilies, easily distinguishable after the phylogeny proposed by Platnick and Forster (1982), the Microstigmatinae Roewer and the Micromygalinae Platnick & Forster (Raven and Platnick 1981; Platnick and Raven 1982).

The subfamily Micromygalinae is characterized by having six spinnerets, a single row of teeth on the paired tarsal claws, flattened tarsal organ, enlarged abdominal scutum in males, serrula teeth clumped into a series, only two eyes, short, slightly produced previous lobe on the palpal coxae, palpal conductor and the loss of book-lungs (Platnick and Raven 1982). The inclusion here of *Tonton* gen. n. within the Micromygalinae expands the generic composition and diversity of this subfamily. The new genus is well supported, especially by the presence of four spinnerets (Fig. 2D), serrula teeth clustered into a series of rows (Fig. 1D), presence of book-lungs (Fig. 2C) and absence of the conductor in the male palp (Fig. 3F).

In addition, *Micromygale* is exclusively distributed within Panama, whereas *Tonton* gen. n. is distributed throughout the Neotropics, though this may be biased due to the absence of more soil sampling in the north of the Americas. This was pointed out by Platnick and Forster (1982) where they express that the fauna of mygalomophs within the leaf litter is still practically unknown. However, recent data published on Masteriinae (Dipluridae) (Passanha and Brescovit 2018) can serve as a comparison, because these tiny spiders have been confused with species of *Tonton* gen. n. in Brazil. The monotypic genus *Micromygale* (with the type species *M.diblemma*) presents a joint distribution with species of *Masteria* in Panama, sharing the distribution of species of Masteriinae (see Passanha and Brescovit, figs 32–33, 39, 45) with *M.downeyi* (Chickering, 1966) and *M.spinosa* (Petrunkevitch, 1925) (Passanha and Brescovit 2018: figs 32, 33). The other species of *Masteria* and species of other genera of Masteriinae are distributed in the Antilles and Amazon region (Almeida et al. 2018, Passanha and Brescovit 2018). In relation to the species within *Tonton* gen. n., all current samples show a disjunctive distribution of the Masteriinae (Fig. 13) with confirmed occurrence in the Brazilian states of northeast and southeast, with many species occurring in natural cavities, despite only one of them being considered a troglobite.

## Supplementary Material

XML Treatment for Tonton

XML Treatment for Tonton itabirito

XML Treatment for Tonton queca

XML Treatment for Tonton matodentro

XML Treatment for Tonton sapalo

XML Treatment for Tonton ipiau

XML Treatment for Tonton quiteria

XML Treatment for Tonton emboaba

## References

[B1] Almeida MQ Salvatierra L Morais JW (2018) A new species of *Masteria* L. Koch, 1873 (Dipluridae: Masteriinae) from Guyana. Zootaxa 4434: 366–368. 10.11646/zootaxa.4434.2.6 30313189

[B2] De Maria M Santos AJ Benedetti AR Faleiro BT Azevedo GH Magalhães IL Pena-Barbosa JPP Santos MT Vilela PF Oliveira U (2009) Aracnídeos/Araneofauna. In: Biodiversidade da Mata Samuel de Paula/AngloGold Ashanti. Belo Horizonte, AngloGold Ashanti Organization, p 209–223.

[B3] Goloboff PA (1989) *Xenonemesia*, un nuevo género de Nemesiidae (Araneae, Mygalomorphae). Journal of Arachnology 16: 357–363.

[B4] Griswold CE (1985) A revision of the African spiders of the family Microstigmatidae (Araneae: Mygalomorphae). Annals of the Natal Museum 27: 1–37.

[B5] Ott R Höfer H (2003) *Enviagarciai*, a new genus and species of mygalomorph spiders (Araneae, Microstigmatidae) from Brazilian Amazonia. Iheringia 93: 373–379. 10.1590/S0073-47212003000400004

[B6] Passanha V Brescovit AD (2018) On the Neotropical spider Subfamily Masteriinae (Araneae, Dipluridae). Zootaxa 4463: 1–73. 10.11646/zootaxa.4463.1.1 30313452

[B7] Pedroso DR Baptista RLC Bertani R (2015) A new species of *Masteria* (Araneae: Dipluridae: Masteriinae) from southeastern Brazil. Zoologia 32: 59–65. 10.1590/S1984-46702015000100009

[B8] Petrunkevitch A (1925) Arachnida from Panama. Transactions of the Connecticut Academy of Arts and Sciences 27: 51–248.

[B9] Platnick NI (2014) The world spider catalog, version 15. American Museum of Natural History. 10.5531/db.iz.0001

[B10] Platnick NI Forster RR (1982) On the Micromygalinae, a new subfamily of mygalomorph spiders (Araneae, Microstigmatidae). American Museum Novitates 2734: 1–13.

[B11] Raven RJ Platnick NI (1981) A revision of the American spiders of the family Microstigmatidae (Araneae, Mygalomorphae). American Museum Novitates 2707: 1–20.

[B12] World Spider Catalog (2018) World Spider Catalog. Natural History Museum Bern. 10.24436/2 [Version 19.0. Accessed on August 28, 2018]

[B13] Wunderlich J (2004) The fossil mygalomorph spiders (Araneae) in Baltic and Dominican amber and about extant members of the family Micromygalidae. Beiträge zur Araneologie 3: 595–631.

